# A multi-objective particle swarm optimization algorithm with two-stage archive maintenance and auxiliary archive guidance

**DOI:** 10.1038/s41598-026-64899-6

**Published:** 2026-08-03

**Authors:** Jing Zhang, Yanmin Liu, Yuci Li, Anna Dai, Ling Zhong, Siwan Chen

**Affiliations:** 1 School of Data Science and Information Engineering, Guizhou Minzu University, Guiyang, 550025 China; 2 School of Mathematics, Zunyi Normal College, Zunyi, 563002 China; 3 School of Mathematics and Statistics, Guizhou University, Guiyang, 550025 China

**Keywords:** Multi-objective particle swarm optimization, Auxiliary archive, Stagnation detection, Adaptive grids, Engineering, Mathematics and computing

## Abstract

In Multi-objective particle swarm optimization (MOPSO), the external archive largely determines how well convergence and diversity are balanced. Ineffective archive maintenance may lead to uneven solution distributions, inaccurate convergence, and premature trapping in local regions.To overcome these limitations, this paper proposes TAMOPSO, a Two-stage Archive Maintenance-based Multi-Objective Particle Swarm Optimization algorithm. In the first stage, adaptive grids with dynamic boundary expansion are used to locate high-density regions. In the second stage, solutions in these regions are evaluated by integrating angle-based diversity assessment and a dual-distance convergence metric, with selection preferences adaptively adjusted according to the evolutionary stage, thereby improving the distribution and Pareto-front coverage of the obtained solution set while controlling archive size. To further enhance particle guidance, a bounded auxiliary archive is introduced to reuse historical high-quality non-dominated solutions discarded during archive maintenance and assist personal best updates. In addition, a stagnation detection-based particle reconstruction strategy is designed, using sparsely distributed elite solutions from the external archive as reconstruction templates to guide stagnant particles back to promising search regions and enhance global exploration. Tests on representative benchmark suites indicate that TAMOPSO produces higher-quality approximation sets than the compared mainstream algorithms in most cases.

## Introduction

Multi-objective optimization problems (MOPs)^1,2^ refer to optimization tasks in which several mutually conflicting objectives need to be optimized simultaneously, and they frequently arise in practical engineering and decision-making scenarios.Different from single-objective optimization, an MOP generally yields a set of acceptable trade-off alternatives rather than one universally best solution. The non-dominated trade-off solutions constitute the Pareto-optimal set^1^, and their objective vectors outline the Pareto front^1^. A solution is Pareto optimal when no feasible move can improve one objective unless at least one remaining objective is worsened.

Owing to their population-based exploration ability, numerous metaheuristics first designed for single-objective tasks have been adapted to the multi-objective setting. Representative examples include the subtr action-averaging optimizer^3^, golden jackal optimization algorithm^4^, bit-linx swarm optimization algorithm^5^, sand cat swarm algorithm^6^, and lens imaging learning arithmetic optimization algorithm^7^. Recently, Zhu et al.^8^ further applied a multi-objective Sand Cat Swarm Optimization variant to containerized microservice deployment, demonstrating the applicability of swarm intelligence algorithms to practical multi-objective engineering problems. Particle swarm optimization (PSO)^9^ has become a widely used swarm-intelligence optimizer for single-objective problems, mainly due to its concise update rules and rapid convergence behavior. The classical PSO framework was later generalized to multi-objective optimization by Coello et al.^10,11^, leading to the early MOPSO paradigm. With its population-based cooperative search strategy, MOPSO has become an effective method for solving MOPs and has been applied to energy management and grid scheduling^12^, manufacturing and operation scheduling^13^, autonomous navigation and path planning for unmanned systems^14^, machine learning and data mining^15^, materials science, and fluid mechanics^16^.

During MOPSO evolution, particles generate candidate solutions repeatedly, while the external archive is refreshed to preserve representative elites. Since the archive can store only a limited number of solutions, the non-dominated candidates generated throughout the search may exceed the available storage space. Therefore, a key challenge is to retain high-quality solutions while preserving the distribution integrity and diversity of the approximated front.

Several strategies have been developed to select non-dominated solutions under finite archive capacity constraints. Early approaches primarily relied on crowding distance^17^, which gives preference to solutions located in less populated regions of the objective space so that the Pareto front can remain well distributed and evenly spread. Adaptive grid methods^18^ divide the objective space into adaptive cells and use the occupancy of these cells as a cue for archive pruning and leader selection. thereby promoting a balanced distribution of solutions across regions. Wang et al.^19^ proposed an adaptive distance mechanism to guide solution selection and retention. Zhang et al.^20^ employed an adaptive projection plane to reduce the objective space with high dimensionality to a two-dimensional representation. Reference points with a uniform distribution were adaptively constructed on this plane, and solution convergence was assessed by associating archived solutions with their nearest reference points, thereby prioritizing solutions in sparse regions.Bai et al.^21^ proposed an evaluation strategy guided by distribution knowledge, introducing a distribution knowledge model to prune solutions with lower contributions based on distribution density, thereby maintaining an efficient external archive.

In recent years, metric-driven archive maintenance strategies have attracted increasing attention. For example, hypervolume-contribution-based strategies prioritize solutions that contribute most to the overall hypervolume during archive updates, thereby improving the hypervolume performance of the archive. Representative algorithms include Supervolume-based Multi-objective Selection (SMS-EMOA)^22^ and the Hypervolume-based Multi-objective Optimization algorithm (HypE)^23^. In addition to hypervolume, Inverted Generational Distance metrics and $\:\epsilon\:$-based indicators are widely employed. Luo et al.^24^ managed external archives by combining angular partitioning with crowding-distance-based screening. Their method partitions the solution space into equal angular regions and removes solutions from overcrowded partitions according to crowding distance while retaining boundary solutions. Zhang et al.^25^ introduced hyper-region density (HRD) in external archive maintenance to characterize the spatial distribution of non-dominated solutions, where lower values indicate denser regions. When the archive overflows, solutions with the smallest HRD values are preferentially deleted, while boundary solutions are preserved with the aid of non-dominated sorting. By replacing traditional crowding distance with hyper-region density, this method mitigates density-estimation bias in high-dimensional spaces and improves archive maintenance accuracy.Cui et al.^26^ employed a dual-archive mechanism combining convergence-oriented archive (CA) and diversity-oriented archive (DA), governed by convergence indicators and density indicators, respectively. The CA selects solutions with superior convergence based on $\:\epsilon\:$-metrics, guiding the swarm toward the true Pareto front. The DA combines offset density estimation with Euclidean distance to preserve boundary solutions while maintaining a uniform solution distribution.Chen et al.^27^ developed a maintenance scheme for the external archive using optimal angular distance. When the archive size exceeds a preset threshold, this strategy uses adaptive grids to identify dense regions and deletes solutions with larger optimal angular distance values within those regions. However, this algorithm depends mainly on density and angular information in the current grid, while failing to fully account for the population’s global evolutionary status. This may result in insufficient preservation of population diversity. Liu et al.^28^ employed a hypercube grid with a two-stage strategy to maintain external archives. Their method first constructs a grid with dynamically adjusted density and then sequentially eliminates high-density and poorly converged solutions using SDE and ASF metrics in two stages. However, with increasing objective dimensionality, hypercube partitioning may become sparsely distributed and imbalanced, leading to less accurate estimation of the solution distribution.

The personal best position ($\:\mathrm{P}\mathrm{b}\mathrm{e}\mathrm{s}\mathrm{t}$) update mechanism exerts a decisive influence on algorithmic performance^29^. In standard MOPSO, $\:\mathrm{P}\mathrm{b}\mathrm{e}\mathrm{s}\mathrm{t}$ updates are typically determined by Pareto dominance relationships. However, this strategy has an inherent limitation: As the population evolves, non-dominated solutions tend to accumulate rapidly.As a result, gradually reduces the ability of Pareto dominance to distinguish among candidate solutions, which may cause $\:\mathrm{P}\mathrm{b}\mathrm{e}\mathrm{s}\mathrm{t}$ updates to stagnate and reduce their ability to guide particle search.

To address search stagnation caused by dominance-based $\:\mathrm{P}\mathrm{b}\mathrm{e}\mathrm{s}\mathrm{t}$ updates in traditional MOPSO, various improvement strategies have been developed. For instance, Kang et al.^30^ replaced the traditional scheme in which each particle independently maintains and updates its own $\:\mathrm{P}\mathrm{b}\mathrm{e}\mathrm{s}\mathrm{t}$. They introduced a socialized competitive framework that distinguishes between winners and losers based on dominance relations, with losers updating their $\:\mathrm{P}\mathrm{b}\mathrm{e}\mathrm{s}\mathrm{t}$ by learning from winners. In this framework, inter-particle competition enables winners to guide losers. Huang et al.^29^ designed a $\:\mathrm{P}\mathrm{b}\mathrm{e}\mathrm{s}\mathrm{t}$ selection mechanism based on memory intervals. Under this strategy, particles maintain a memory interval rather than a single $\:\mathrm{P}\mathrm{b}\mathrm{e}\mathrm{s}\mathrm{t}$. This interval preserves a sequence of non-dominated solutions discovered during recent iterations, serving as a candidate set for $\:\mathrm{P}\mathrm{b}\mathrm{e}\mathrm{s}\mathrm{t}$. Wang et al.^31^ employed an adaptive PBI distance policy and simplified position update mechanism to select superior leader particles from the elite population. However, this algorithm relies mainly on the current elite population as the guidance source and neglects valuable historical information. Li et al.^32,33^ introduced a competition mechanism and structured spatial partitioning to promote local competitive learning, enabling unsuccessful particles to learn from successful ones. However, this approach neglects each particle’s self-experience-based cognitive guidance and useful information beyond the winner particles.

Moreover, particles may be confined to local optimal regions or exhibit insufficient learning capabilities during the search process. If these particles are not identified and improved in time, the algorithm’s overall effectiveness may be degraded. To address this issue, Zhang et al.^20^ applied Cauchy mutation to the densest particles when the population distribution became excessively crowded, encouraging them to migrate toward sparser regions. However, this operation is essentially an unguided random perturbation and lacks intelligent guidance. It does not distinguish whether the mutated particles are superior or inferior. Shu et al.^34^ enhanced the learning mechanism by dynamically adjusting learning targets according to particle distribution, thereby preventing blind following and lowering the likelihood that particles are confined to local optima. Other methods, including CDA-MOPSO^35^, ADN-MOPSO^36^, and MOCPSO^37^, have also been proposed to avoid local optima.

Although these methods have significantly advanced archive management and selection strategies, their emphasis is still largely placed on the solutions preserved in the external archive. By contrast, the useful information contained in discarded non-dominated solutions has received limited attention, and evolutionary stagnation at the particle level remains insufficiently handled. In contrast, the proposed TAMOPSO not only adopts a two-stage archive maintenance strategy but also introduces a bounded auxiliary archive to reuse discarded non-dominated solutions for guiding *\:pbest* updates, and further incorporates a stagnation-detection-based particle reconstruction mechanism.Therefore, TAMOPSO establishes a unified framework that integrates archive update, historical solution reuse, and particle-level regeneration to enhance both exploration and exploitation capabilities.

The key contributions of TAMOPSO are outlined below:

- External archive updates based on a two-phase archive maintenance scheme: In the initial phase, an adaptive grid technique with dynamic boundary expansion is introduced.This strategy adaptively locates high-density regions using an expansion coefficient related to both the iteration number as well as the dispersion degree of the solution set. In the subsequent phase, local elite selection is performed within the identified high-density regions. A composite ranking formula is designed by adaptively weighting minimum-angle-based diversity evaluation and dual-distance convergence metrics based on the minimum-value and maximum-value reference points. This formula guides archive insertion and deletion during the iterative process.
- An auxiliary-archive-assisted $\:\mathrm{p}\mathrm{b}\mathrm{e}\mathrm{s}\mathrm{t}$ update mechanism: This mechanism gathers valuable historical non-dominated solutions removed during archive maintenance and constructs a bounded auxiliary archive to support $\:\mathrm{p}\mathrm{b}\mathrm{e}\mathrm{s}\mathrm{t}$ updates. By integrating multi-objective improvement indicators, weighted fusion, and historical perturbation, this mechanism suppresses particle stagnation in unproductive regions and improves both diversity and guidance effectiveness.
- A stagnation-detection-based particle reconstruction: A particle flight stagnation detector was developed.Guided by sparsely distributed elite solutions from the external archive, this strategy guides poorly performing particles out of local optimal regions and moves them toward more promising areas of the search space.

The rest of this paper is structured as follows. Section 2 presents the basic concepts of MOP, PSO and MOPSO. Section 3 describes the TAMOPSO in detail, including the two-stage archive maintenance strategy, the auxiliary-archive-driven pbest update mechanism, and the stagnation-detection-based particle reconstruction strategy.Sect. 4 reports the comparative experimental results and analyzes the performance of TAMOPSO. Section 5 provides the conclusions, discusses its limitations, and suggests possible directions for future work.

## Foundations of multi-objective optimization and particle swarm optimization

### Definition of multi-objective optimization

Multi-objective optimization problems refer to mathematical problems requiring the simultaneous optimization of multiple conflicting objectives, involving two or more optimization goals^1,2^. For instance: minimizing cost whilst maximizing performance. Taking objective minimization as an example, its mathematical formulation is:

1 $$\begin{array}{*{20}c} {Min} & {F\left( x \right) = \left[ {f_{1} \left( x \right),f_{2} \left( x \right),...,f_{M} \left( x \right)} \right]} \\ {Subject~to} & {g_{i} \left( x \right) \le 0,~~i = 1,2,...,q} \\ {\,\,} & {h_{j} \left( x \right) = 0,~~~j = 1,2,...,q} \\ \end{array}$$

Where *\:x* denotes the decision variable vector, $\:\mathrm{F}\left(\mathrm{x}\right)$ represents the objective function vector, $\:{\mathrm{g}}_{\mathrm{i}}\left(\mathrm{x}\right)$ denotes the inequality constraints, and $\:{\mathrm{h}}_{\mathrm{j}}\left(\mathrm{x}\right)$ denotes the equality constraints. A constraint in the form$\:\:{\mathrm{g}}_{\mathrm{i}}\left(\mathrm{x}\right)\ge\:0$ can be equivalently transformed into $\:-{\mathrm{g}}_{\mathrm{i}}\left(\mathrm{x}\right)\le\:0$.

### Particle swarm optimization

Within the PSO framework, particles represent candidate solutions to an optimization problem, characterized by two state variables, namely position and velocity^9^. These variables are updated through iterative updates under the guidance of the particle’s personal best position and the swarm-level best position, gradually approach an optimal region.

The velocity of particle *i* at iteration *\:t+1* is calculated as follows:

2 $$\:{v}_{i}^{t+1}=\omega\:\cdot\:{v}_{i}^{t}+{c}_{1}\cdot\:{r}_{1}\cdot\:({pbest}_{i}-{x}_{i}^{t})+{c}_{2}\cdot\:{r}_{2}\cdot\:({gbest}_{i}-{x}_{i}^{t})$$

Where $\:{v}_{i}^{t+1}$ represents the velocity of particle *i* at time *\:t*+1, and $\:{v}_{i}^{t}$ represents the particle’s current velocity at time t. $\:\omega\:$ is the inertia weight, determines the extent to which the previous velocity is preserved.$\:{c}_{1}$ and $\:{c}_{2}$ are acceleration coefficients, used to balance the influence of individual experience and group experience respectively. $\:{pbest}_{i}$ signifies the best position previously found by particle *i*, $\:{gbest}_{i}$ denotes the best position discovered by the swarm, and $\:{x}_{i}^{t}$ is the current position of particle *i* at iteration *\:t*.

The position update is then performed as follows:

3 $$\:{x}_{i}^{t+1}={x}_{i}^{t}+{v}_{i}^{t+1}$$

### Multi-objective particle swarm optimization

Multi-objective particle swarm optimization (MOPSO) adapts the conventional PSO framework for multi-objective problems by integrating Pareto dominance with an external archive^10,11^. Unlike single-objective PSO, MOPSO seeks to generate a diverse set of non-dominated solutions instead of identifying only one optimum. In a minimization problem, solution $\:\mathrm{x}$ dominates solution $\:\mathrm{y}$ if $\:{\mathrm{f}}_{\mathrm{m}}\left(\mathrm{x}\right)\le\:{\mathrm{f}}_{\mathrm{m}}\left(\mathrm{y}\right)$ for all objectives and $\:{\mathrm{f}}_{\mathrm{m}}\left(\mathrm{x}\right)<{\mathrm{f}}_{\mathrm{m}}\left(\mathrm{y}\right)$ for at least one objective.i.e.,$x \prec y \Leftrightarrow \left( {\forall m,f_{m} \left( x \right) \le f_{m} \left( y \right)} \right) \wedge \left( {\exists m,f_{m} \left( x \right) < f_{m} \left( y \right)} \right),$ $\:\mathrm{A}\mathrm{r}\mathrm{c}=\left\{\left.\mathrm{x}\in\:{\mathrm{P}}_{\mathrm{t}}\cup\:\mathrm{A}\mathrm{r}\mathrm{c}\right|\nexists\:\mathrm{y}\in\:{\mathrm{P}}_{\mathrm{t}}\cup\:\mathrm{A}\mathrm{r}\mathrm{c},\mathrm{y}\prec\:\mathrm{x}\right\}$. During evolution, the external archive $\:\mathrm{A}\mathrm{r}\mathrm{c}$ is used to store non-dominated solutions produced during population evolution. The archive provides the final Pareto-front approximation and also supplies candidate solutions for global guide selection. The velocity and position of each particle are updated under the guidance of its personal best $\:\mathrm{p}\mathrm{b}\mathrm{e}\mathrm{s}\mathrm{t}$ and a global guide chosen from $\:\mathrm{A}\mathrm{r}\mathrm{c}$.

### The proposed TAMOPSO algorithm

**Fig. 1:**
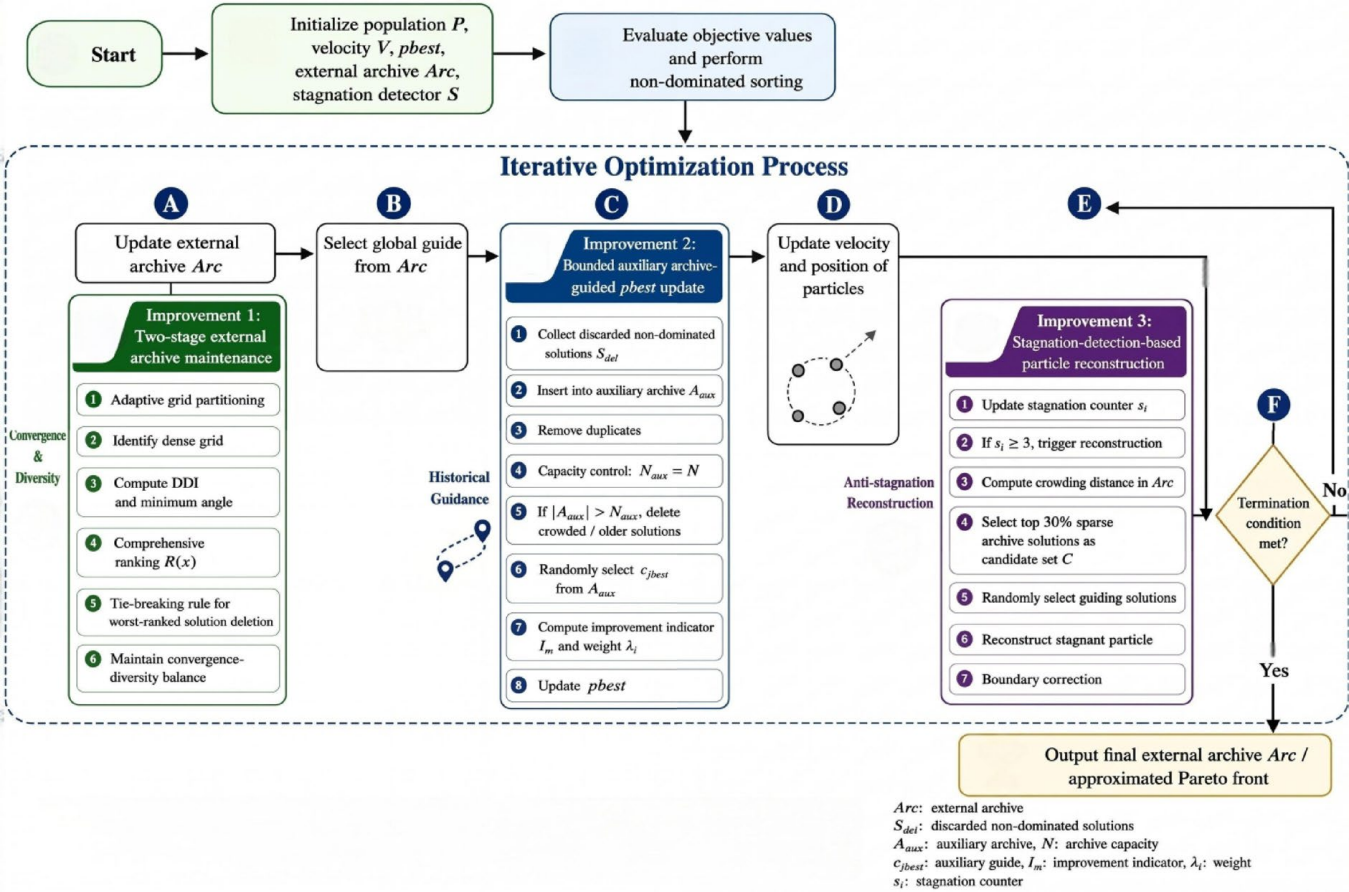
Graphical framework of TAMOPSO.

TAMOPSO begins by initializing the particle population, velocity, personal best positions, external archive, and stagnation detector. During each iteration, the external archive is first updated using the proposed two-stage archive maintenance strategy to preserve high-quality non-dominated solutions while controlling archive size. Subsequently, historical non-dominated solutions discarded during archive maintenance are collected into the bounded auxiliary archive and used to assist the personal-best update. After particle velocities and positions have been renewed, stagnant particles are detected and reconstructed using sparsely distributed elite solutions selected from the external archive. This iterative process proceeds until the stopping criterion is met, and the final external archive is returned as an approximation of the Pareto front. Figure 1 presents the overall workflow of TAMOPSO. A general pseudocode is provided in Algorithm 1.

**Algorithm 1:**
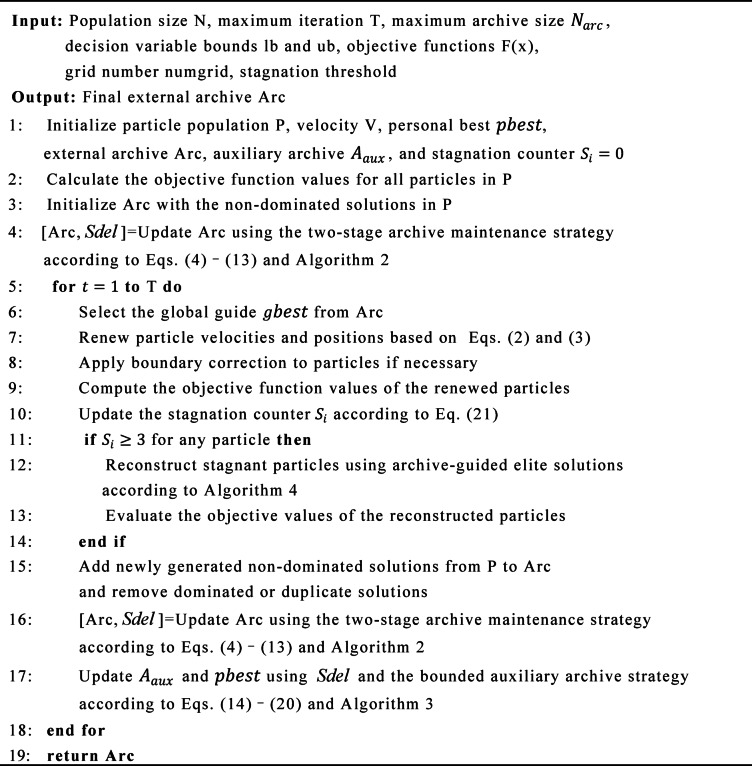
Overall procedure of TAMOPSO.

### External archive update based on two-stage maintenance strategy

In multi-objective particle swarm optimization algorithms, an external archive is commonly maintained for preserving non-dominated solutions obtained throughout the search process. This archive provides candidate guide solutions for determining gbest during particle movement but also represents the solution set returned at the end of the algorithm. To control storage scale, a preset archive capacity threshold is generally implemented. However, as iterations progress, the population may produce numerous non-dominated candidates as the search proceeds. Therefore, an efficient archive maintenance mechanism is required to retain solutions with good diversity and convergence properties while eliminating poorly performing ones, thereby achieving effective management of archive resources. Existing methods predominantly rely on density estimation for archive maintenance, favoring the removal of particles from high-density regions within the objective space. Common density evaluation strategies include adaptive grid methods, crowding distance, and angle-based approaches. However, relying solely on a single density metric often fails to comprehensively assess particle diversity. Furthermore, among the non-dominated candidate solutions in high-density regions, comprehensively evaluating both particle convergence and distribution remains challenging.

To overcome the above limitations, a two-phase archive maintenance strategy is designed in this study: the first stage accurately identifies high-density regions by constructing adaptive grids; the second stage comprehensively evaluates and selects particle performance within these regions by integrating diversity and convergence metrics. The specific operational process is as follows:

During iteration, once the archive contains more non-dominated candidates than its predefined capacity, the proposed two-phase maintenance procedure is activated.The initial phase mainly involves constructing adaptive grids and assigning particles to corresponding grid cells, following the steps below: For each objective dimension, if all objective values for that dimension are equal, only two boundary grid lines are set: 0 and the constant objective value. Where target values differ, the dimension is uniformly partitioned into 49 intervals, generating 50 equidistant grid lines. To prevent allocation ambiguities caused by particles falling on grid boundaries, a dynamic boundary expansion coefficient *\:ep* is introduced during grid line configuration, defined as:

4 $$\:ep=0.05\cdot\:(1+\frac{t}{T})\cdot\:{\sigma\:}_{f}$$

Here, t denotes current iteration number, T denotes maximum number of iterations,$\:{{\upsigma\:}}_{\mathrm{f}}$ represents the standard deviation of objective values along the current dimension, effectively gauging the dispersion of solutions. A larger $\:{{\upsigma\:}}_{\mathrm{f}}$ value indicates solutions are widely dispersed along that dimension, warranting a larger *\:ep* value to match the actual distribution range and ensure boundary expansion is ‘proportionate’. Conversely, a smaller $\:{\sigma\:}_{f}$ value signifies solutions are concentrated along that dimension (potentially converging within a narrow interval), where a smaller *\:ep* value suffices, employing $\:{\sigma\:}_{f}$ enables the mesh generation to better conform to the actual distribution of solutions.

The grid spacing is defined as:

5 $$tempf = \frac{{f_{{\max }} (k) + f_{{\min }} (k) + 2ep}}{{numgrid - 1}}$$

Where $f_{{\max }} (k)$ and $f_{{\min }} (k)$ denote the maximum and minimum objective function values in the k-th dimension.In the formula, *numgrid* equals 50. Based on this spacing, the second grid line is positioned at *tempf* to the right of the first grid line.Subsequent grid lines follow this pattern, with the final grid line located at $\:{f}_{max}\left(k\right)+ep.$ After completing the construction of grid lines across all dimensions, proceed to the particle grid positioning stage. By calculating the Euclidean distance *d* between each non-dominated solution and the grid lines of each dimension, the corresponding grid is determined. $\:{f}_{ij}$ represents the fitness value of the i-th particle on the j-th dimension; $\:{f}_{{grid}_{j,k}}$ stands for the coordinate value of the k-th grid line on the j-th dimension. ${d^{\prime}}$ is calculated as half the distance between two adjacent grid lines on each dimension plus a minor offset. If this distance is less than ${d^{\prime}}$, the particle is assigned to the current grid. The two distances are defined as follows:

6 $$d = dist(f_{{ij}} ,fgrid_{{j,k}} )$$

7 $$d^{\prime} = \frac{{fgrid_{{j,2}} - fgrid_{{j,1}} }}{2} + \frac{{fgrid_{{j,2}} - fgrid_{{j,1}} }}{{1000}}$$

 

This criterion balances reasonable assignment margin with numerical stability, ensuring each non-dominated candidate can be precisely assigned to the appropriate grid cell. This lays the foundation for subsequent identification of high-density regions.

After completing the grid positioning for all non-dominated solutions, count the number of solutions contained within each grid. Define the grid containing the highest number of non-dominated candidates as a high-density region. Subsequently, proceed to the second phase of the maintenance mechanism, conducting a detailed analysis of the solutions within this high-density region to eliminate individuals with poorer overall performance. Specifically, high-density regions selected through the adaptive grid strategy in the first phase typically indicate relatively insufficient diversity among solutions within that area. To achieve more precise filtering under these conditions, both diversity and convergence of the solutions in this region need to be jointly assessed. This paper employs a diversity assessment method based on the angle strategy, calculated by determining the minimum angle $\:{\theta\:}_{i,min}$ between solutions within the region.

8 $$\theta _{{i,\min }} = \min (\theta _{{i.j}} )$$

 

9 $$\theta _{{i,j}} = \arccos \frac{{\left| {\sum\nolimits_{{m = 1}}^{M} {f_{{i,m}} f_{{j,m}} } } \right|}}{{\sqrt {\sum\nolimits_{{m = 1}}^{M} {f_{{i,m}}^{2} } \sqrt {\sum\nolimits_{{m = 1}}^{M} {f_{{j,m}}^{2} } } } }}$$

where $\mathrm{M}$ denotes the number of objective dimensions of the test function.$\:{\mathrm{f}}_{\mathrm{i},\mathrm{m}}$ represents the fitness value of the i-th particle on the m-th objective dimension of the test function.

After completing the angle assessment, particles are ranked based on their minimum angle value $\:{{\uptheta\:}}_{\mathrm{i},\mathrm{m}\mathrm{i}\mathrm{n}}$. Since a larger minimum angle value indicates that the particle maintains better distribution characteristics in its surroundings, reflecting superior diversity quality, the solution with the largest $\:{{\uptheta\:}}_{\mathrm{i},\mathrm{m}\mathrm{i}\mathrm{n}}$ is given the best ranking, namely Rank1.

In convergence assessment, convergence quality is typically defined as the proximity of the population to the true Pareto front. A common way to assess convergence is to calculate the Euclidean distance from a solution to the ideal point; however, this measure can be strongly affected by the geometry of the objective space. For instance, within convex objective function regions, the inherent bias toward ideal points causes individuals near the center of the objective space to receive disproportionate favor, thereby reducing convergence pressure. Specifically, when two individuals located in different directions share equal distances from the ideal point, the ideal point distance alone proves insufficient to effectively distinguish their convergence quality.This paper employs a dual-distance convergence metric based on MDCS^19^, specifically: First, the distances between each non-dominated candidate in the selected region and the minimum-value and maximum-value reference points are computed.

10 $$d_{{i,1}} = \sqrt {\sum\nolimits_{{m = 1}}^{M} {(f_{{i,m}} - z_{m}^{*} )^{2} } }$$

11 $$d_{{i,2}} = \sqrt {\sum\nolimits_{{m = 1}}^{M} {(f_{{i,m}} - z_{m}^{{\max }} )^{2} } }$$

Where $\:{\mathrm{z}}_{\mathrm{m}}^{\ast\:}$ denotes the minimum value of the m-th objective among all candidate solutions in archive, while $\:{\mathrm{z}}_{\mathrm{m}}^{\mathrm{m}\mathrm{a}\mathrm{x}}$ denotes the maximum value of the m-th objective among all candidate solutions in archive.

Next, integrate the above two formulas to establish a unified metric:

12 $$DDI_{i} = \max (d_{{1,i}} - d_{{1,\min }} ,d_{{2,\max }} - d_{{2,i}} )$$

The above formula integrates the proximity of an individual to the reference point formed by the minimum objective values and its relative distance to the reference point formed by the maximum objective values into a unified minimization problem. A smaller DDI value indicates that the individual exhibits better overall convergence within the current region. Therefore, in convergence ranking, the individual with the smallest DDI value is assigned the highest rank, namely rank 1. Unlike existing convergence metrics that evaluate convergence from a single direction or rely only on objective-difference preferences, this metric simultaneously considers both the minimum-value reference point and the maximum-value reference point in the current candidate solution set. By introducing reference information from both sides of the objective space, the proposed metric can provide more balanced selection pressure and avoid concentrating the selected solutions only around the minimum-value reference point.This mechanism improves the algorithm’s ability to handle complex Pareto-front structures.

**Fig. 2:**
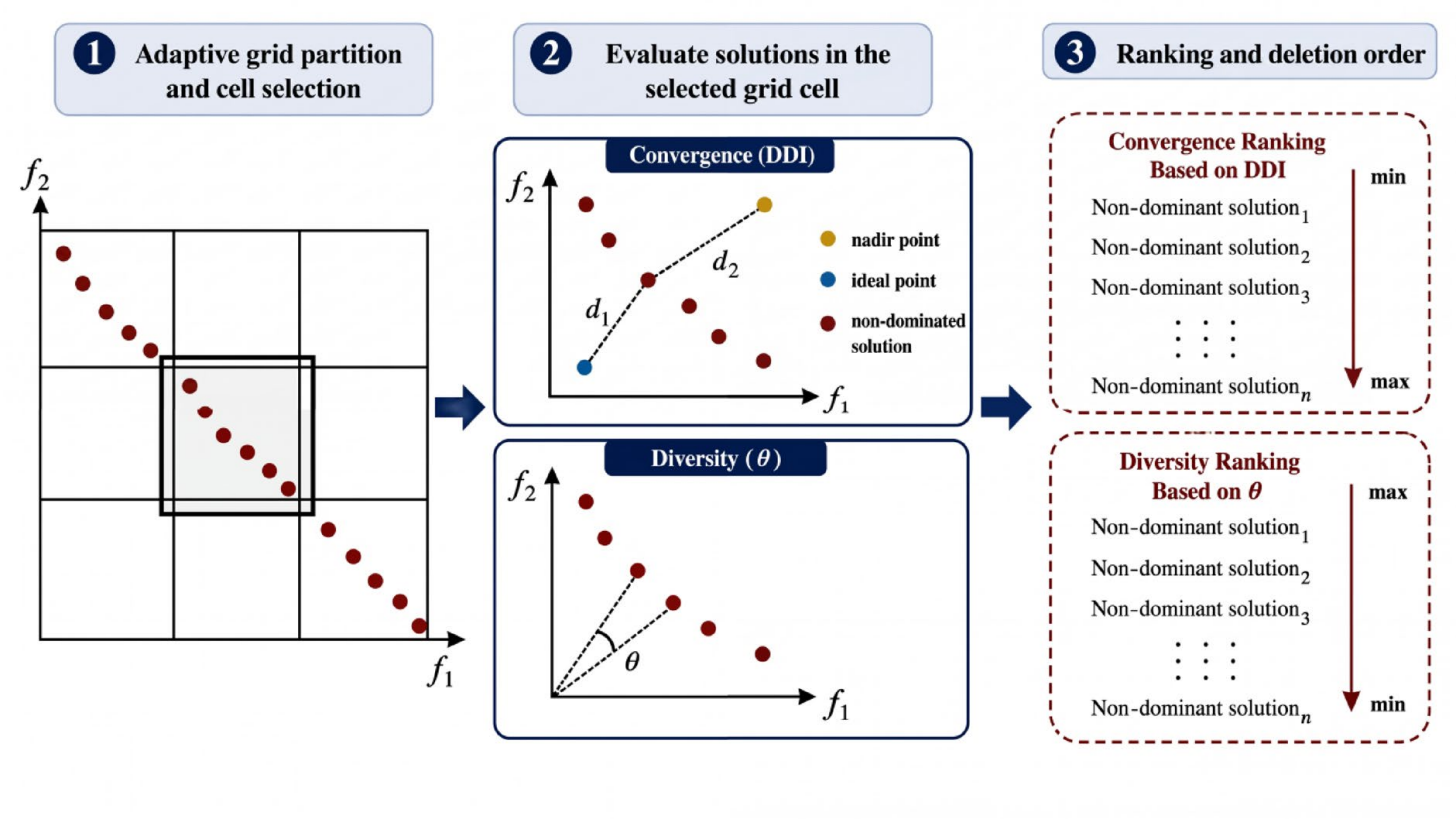
The principle of non-dominated deletion.

After obtaining diversity rankings and convergence rankings, these two metrics are integrated to form a unified assessment of solutions within high-density regions.Considering that algorithms should prioritize different aspects of diversity and convergence at different evolutionary stages—specifically, enhancing convergence should be prioritized in the early iterations while maintaining diversity becomes crucial in later stages—this paper proposes the following integrated ranking evaluation method:

13 $$\:R\left(x\right)=(1-\frac{t}{T})Crank\left(x\right)+\frac{t}{T}Drank\left(x\right)$$

In this study, the evolutionary stage is defined by the normalized iteration ratio $$\:\raisebox{1ex}{$t$}\!\left/\:\!\raisebox{-1ex}{$T$}\right.$$.where *\:x* denotes a solution in archive, $$\:R\left(x\right)$$ denotes the comprehensive ranking score of solution *\:x*, $$\:Crank\left(x\right)$$ indicates the convergence ranking of solution *\:x* obtained according to the DDI metric, while *Drank(**x**)* denotes the diversity ranking of solution *\:x* obtained according to the minimum angle. Since Rank1 represents the best performance in both convergence and diversity rankings, a smaller $$\:R\left(x\right)$$ indicates better overall performance, whereas a larger $$\:R\left(x\right)$$ indicates poorer overall performance.

Since both *Crank(**\:x**)* and *Drank(**\:x**)* are discrete ranking values, several solutions may obtain identical comprehensive ranking scores in Eq. (13). To avoid ambiguous deletion decisions, a deterministic tie-breaking rule is introduced. Let $\:\varOmega\:$ denote the set of candidate solutions with the worst comprehensive ranking score, namely $\:\varOmega\:=\left\{x\in\:Archive\left|R\left(x\right)=\right.maxR\left(y\right),y\in\:Archive\right\}$. If $\:\left|\varOmega\:\right|=1$, the solution in $\:\varOmega\:$ is directly removed. Otherwise, the secondary criterion is selected based on the current stage of evolution. At the early phase, i.e.,$\:\frac{t}{T}<0.5$, convergence is assigned a higher priority; therefore, the solution with the largest DDI value in $\:\varOmega\:$ is removed. If several solutions still have the same DDI value, the candidate solution having the lowest minimum-angle value is deleted.In the later stage, i.e., $\:\frac{t}{T}\ge\:0.5$, diversity preservation becomes more important; therefore, the candidate in $\:\varOmega\:$ with the lowest minimum-angle value is removed first. If a tie still exists, the solution with the largest *DDI* value is removed. Finally, if the above criteria still cannot distinguish the candidates, the candidate solution with the lowest local crowding-distance value is eliminated. Random deletion is performed only when all the above values are identical within a predefined numerical tolerance. This rule makes the archive maintenance process deterministic in most cases and ensures that the deletion decision remains consistent with the convergence-diversity preference of Eq. (13).

Based on the comprehensive ranking results, the lowest-ranked particle is eliminated.Subsequently, the archive size is evaluated against the preset threshold requirement. If the threshold remains unmet, high-density regions within the current archive are re-identified, and the evaluation and elimination process from the second phase is repeated until the archive capacity falls within the specified range.

To clearly illustrate the workflow of the two-stage archive maintenance mechanism, an illustrative example based on a two-objective optimization problem is provided, and its solution distribution is shown in Fig. 2. The current archive contains 16 non-dominated solutions, while the preset capacity threshold is set at 15. The maintenance process first employs adaptive mesh refinement to identify the grid cell with the largest number of non-dominated candidates, namely the most crowded region. Subsequently, a second-order comprehensive performance evaluation is conducted on all solutions within this region, ultimately eliminating the solution with the poorest overall performance. The specific implementation of this section’s strategy is detailed in Algorithm 2.

**Algorithm 2:**
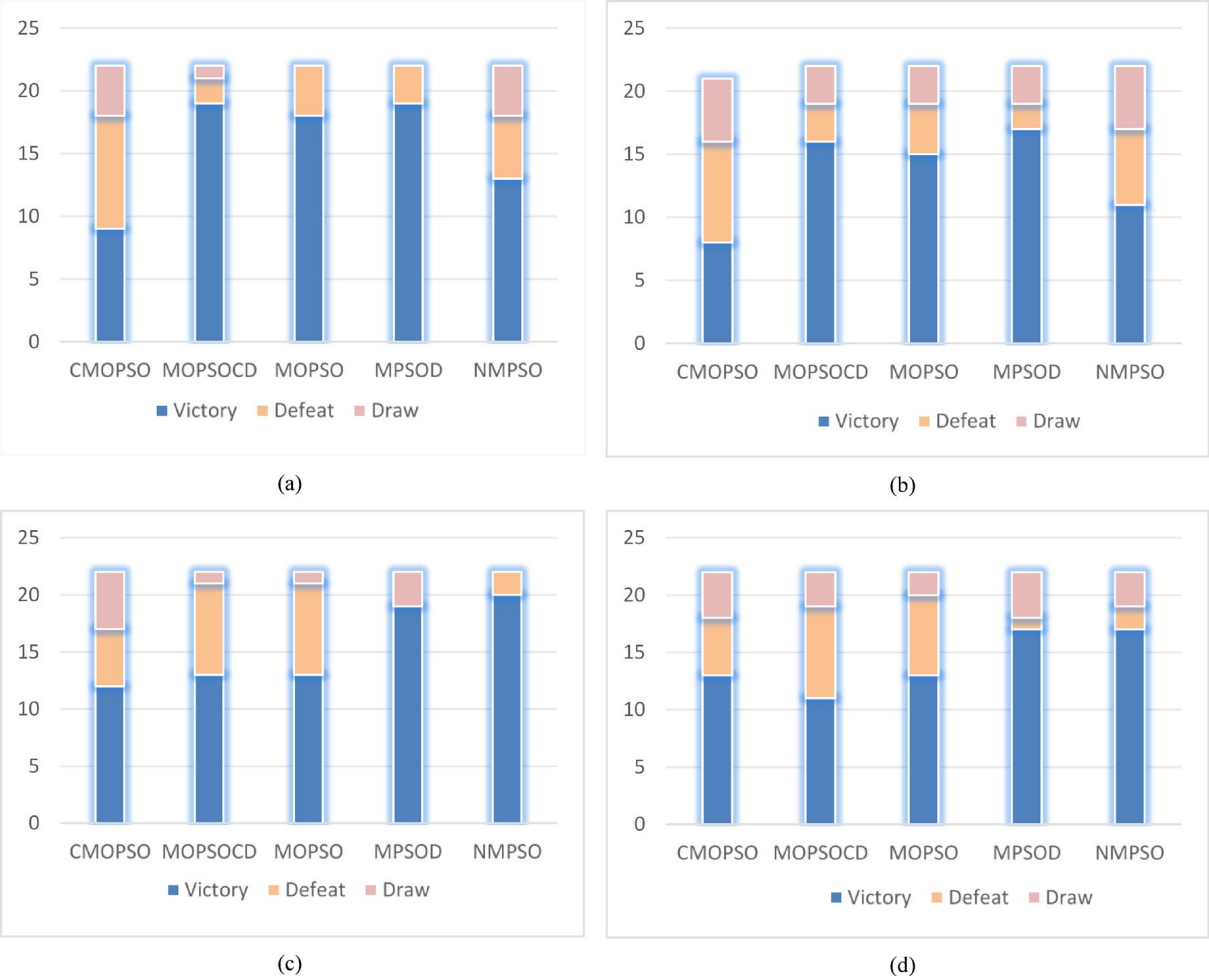

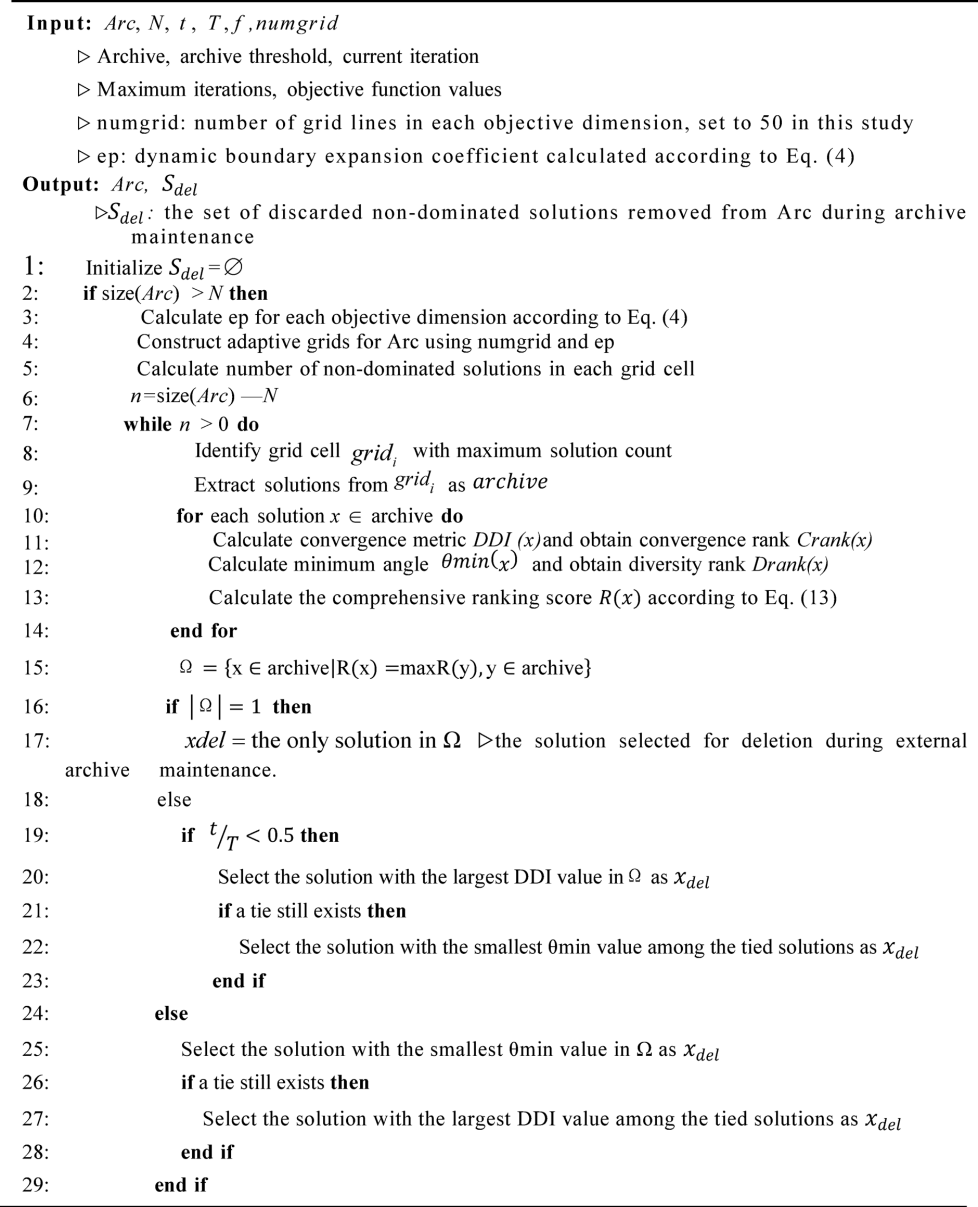
External archive update based on two-stage maintenance strategy.

### $\:\mathbf{P}\mathbf{b}\mathbf{e}\mathbf{s}\mathbf{t}$ update based on bounded auxiliary archive

In the MOPSO, the best individual is essential for guiding the search process. It serves as the foundational memory guiding the population’s flight to cover the entire Pareto front, while also being a crucial component in maintaining population diversity. Specifically, during the velocity update, the particles learn towards their own *\:Pbest* direction. This mechanism ensures that particles tend to fly towards previously discovered high-quality regions, acting as a key driving force for algorithmic convergence. The diversity of *\:Pbest* fosters diversity in search directions, as different particles’ *\:Pbest* may be distributed across distinct regions of the Pareto front, particles learning towards their pbest are effectively guided to explore different regions of the front. This distributed exploration mechanism helps the algorithm cover the Pareto front more effectively while preserving population diversity. Given the pivotal role of the *\:Pbest*, the design of its update strategy becomes a core factor influencing the performance of the MOPSO algorithm, with different strategies directly impacting its efficacy.

**Fig. 3:**
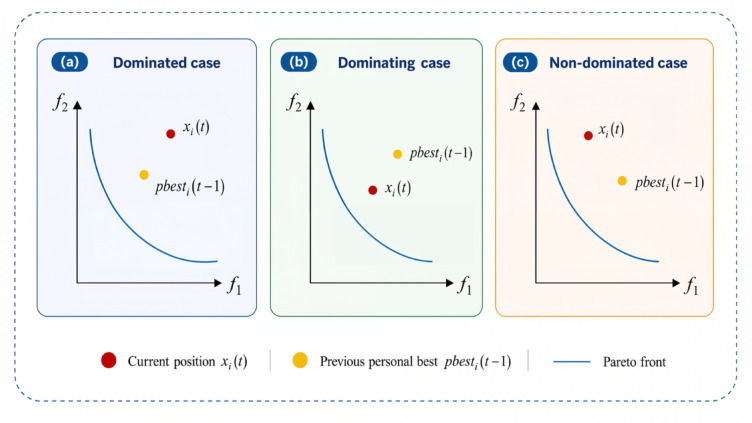
Three typical scenarios that may be encountered during the *\:Pbest* update process.

Traditional *\:Pbest* updates employ a dominance-based approach: if a new position dominates the original *\:Pbest*, it replaces the current position; if the original *\:Pbest* dominates the new position, it remains unchanged; if neither solution dominates the other, one of them is chosen at random as the new *\:Pbest*. However, this mechanism has significant shortcomings. Figure 3 presents three representative situations that may occur during *\:Pbest* updating. In Fig. 3a, the current position is still guided by the previous *\:Pbest*. Under conventional update principles, *\:Pbest* remains unchanged, continuing to utilize the original position. However, under the guidance of the original *\:Pbest*, the particle flight exhibited degradation. Persisting with sub-optimal *\:Pbest* causes particles to deviate further from the Pareto front. Figure 3b shows that the current iteration of the particle dominates the original *\:Pbest*. Under the guidance of the original *\:Pbest*, the particles exhibit an evolutionary state. However, if the latest position replaces *\:Pbest*,the cognitive term in the particle flight update formula becomes zero, thereby generating meaningless.

Individual optimal values during the flight process.At this point, the algorithm primarily relies on the global optimum (*\:gbest*) for driving, weakening its fine-grained search capability based on local information. Figure 3c depicts a scenario in which neither position dominates the other, resulting in particle stagnation. An analysis of the above circumstances reveals that whichever option is selected as *\:Pbest* carries inherent shortcomings. To improve*\:Pbest* updating, a collaborative update mechanism supported by auxiliary archives is developed in this study.

As previously stated, the algorithm may produce many non-dominated candidates at each iteration; some are inserted into the external archive, whereas others are removed. Furthermore, existing solutions within the archive may be replaced during the maintenance phase. Consequently, this study constructed an auxiliary archive.

Solutions within auxiliary archives originate from non-dominated solutions generated during the current or previous iteration. Although these solutions are not retained in external archives, they remain high-quality solutions in the population, particularly in the early iterations. They may harbor valuable historical information that is otherwise discarded during external archive maintenance, potentially leading to possible information loss. To address this issue, this study proposes the reuse of this information by integrating it into the *\:Pbest* update process to enhance algorithmic performance.

**Fig. 4:**
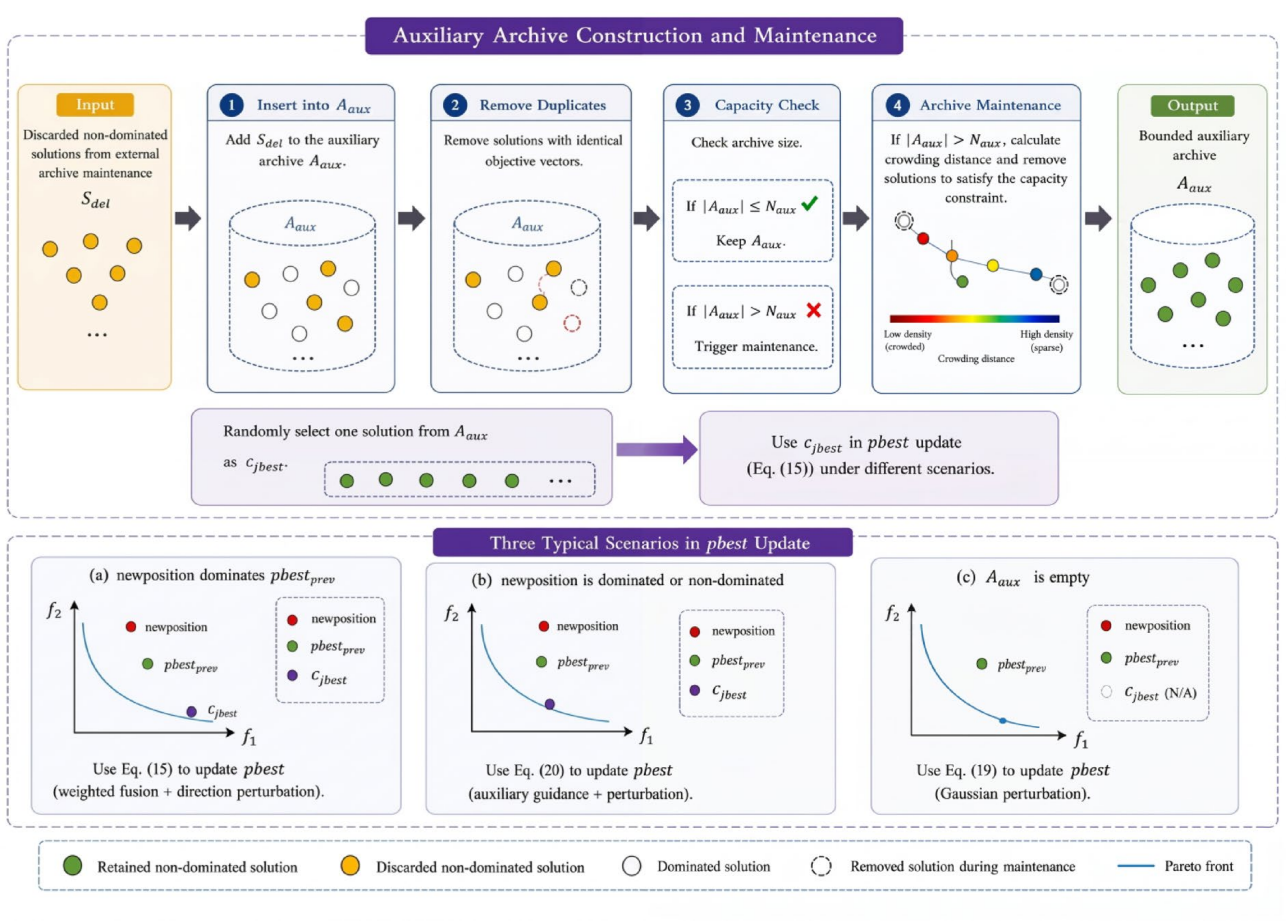
Construction, maintenance, and utilization mechanism of the bounded auxiliary archive.

Figure 4 presents the construction process of the auxiliary archive. Let $\:{S}_{del}$ denote the set of non-dominated solutions discarded during the external archive maintenance process owing to the limited capacity of *Arc*. Although these solutions are not retained in *Arc*, they still contain useful historical search information and are therefore inserted into the auxiliary archive $\:{A}_{aux}$.

Specifically, $\:{S}_{del}$ is generated when the external archive exceeds its capacity threshold during the archive maintenance process. The solutions selected for deletion $\:{x}_{del}$ are first removed from Arc and then stored in $\:{S}_{del}.$ Therefore, $\:{S}_{del}$ serves as an intermediate solution set connecting the external archive maintenance procedure and the bounded auxiliary archive mechanism. The solutions contained in $\:{S}_{del}$ are subsequently transferred to the auxiliary archive update procedure for further utilization.

To prevent the auxiliary archive from expanding without restriction, a bounded auxiliary archive is introduced. Let $\:{A}_{aux}$ denote the auxiliary archive, and let $\:{N}_{aux}$ denote its maximum capacity. To avoid introducing an additional empirical parameter, $\:{N}_{aux}$ is defined as the same value as the capacity threshold N of the external archive, i.e., $\:{N}_{aux}=N$. This setting keeps the auxiliary archive at the same scale as the current swarm. If the auxiliary archive is too small, useful historical non-dominated solutions may be lost and the auxiliary guidance for $\:\mathrm{p}\mathrm{b}\mathrm{e}\mathrm{s}\mathrm{t}$ updating may become insufficient. In contrast, an excessively large auxiliary archive may introduce redundant historical information and raise the computational burden of archive maintenance.

Figure 4 illustrates the construction, maintenance, and utilization mechanism of the bounded auxiliary archive. The historical non-dominated candidates removed during archive maintenance are are first gathered into $\:{S}_{del}$ and inserted into the auxiliary archive $\:{A}_{aux}$. To avoid redundant historical information, duplicate solutions are removed according to their objective vectors and decision vectors. Then, the size of $\:{A}_{aux}$ is checked against its maximum capacity $\:{N}_{aux}$. If $\:\left|{A}_{aux}\right|\le\:{N}_{aux}$, the auxiliary archive is retained without further deletion; otherwise, archive maintenance is triggered. Specifically, the crowding-distance value for each solution in $\:{A}_{aux}$ is computed, and the solution with the smallest crowding distance is removed first to satisfy the capacity constraint. Through this process, a bounded auxiliary archive is obtained.

After archive maintenance, one solution $\:{\mathrm{c}}_{\mathrm{j}}\mathrm{best\:}$is randomly selected from $\:{A}_{aux}$ and used to assist the $\:\mathrm{pbest}$ update under different situations.

Based on the bounded auxiliary archive, the $\:\mathrm{pbest}$ update strategy is redesigned according to the dominance relationship between the current position and the previous personal best. Let$\:{\mathrm{\:newposition}}_{i}\:$ denote the current position of the *i-th* particle, and let $\:{\mathrm{pbest}}_{i}^{\:prev}$ denote its previous personal best. The auxiliary archive $\:{A}_{aux}$ provides historical non-dominated information for $\:\mathrm{pbest\:}$ update. When $\:{A}_{aux}$ is not empty, a solution $\:{\mathrm{c}}_{\mathrm{j}}\mathrm{best}$ is randomly selected from $\:{A}_{aux}$ as the auxiliary guidance solution.

14 $$\:{\mathrm{pbest}}_{i}^{t+1}\mathrm{=}{\mathrm{newposition}}_{i}$$

If $\:{\mathrm{\:newposition}}_{i}$dominates $\:{\mathrm{pbest}}_{i}^{\:prev}$ and $\:{A}_{aux}$ is empty, no auxiliary historical information is available. Therefore, the personal best is directly updated by the current position according to Eq. (14).

15 $$pbest_{i}^{{t + 1}} = \lambda _{i} \cdot newposition_{i} + (1 - \lambda _{i} )c_{j} best + \beta _{i} \cdot(newposition_{i} \,0\,pbest_{i}^{{prev}} )$$

If $\:{\mathrm{\:newposition}}_{i}$ dominates $\:{\mathrm{pbest}}_{i}^{\:prev}$ and $\:{A}_{aux}$ is not empty, directly replacing $\:{\mathrm{pbest}}_{i}^{\:prev}$ with ${\mathrm{newposition}}_{i}$ may weaken the cognitive component in the next velocity update. Therefore, a historical solution$\:{\mathrm{c}}_{\mathrm{j}}\mathrm{best}$ is randomly selected from $\:{A}_{aux}$ and combined with ${\mathrm{newposition}}_{i}$ In Eq. (15), the first term inherits information from the current improved position, the second term introduces historical guidance from the auxiliary archive, and the third term preserves the improvement direction from $\:{\mathrm{pbest}}_{i}^{\:prev}$ to ${\mathrm{newposition}}_{i}$.

16 $$\:{{\upbeta\:}}_{\mathrm{i}}={r}_{i}(1-\frac{\mathrm{t}}{\mathrm{T}}),\:\:\:\:{r}_{i}\sim\:\cup\:\left(\mathrm{0,1}\right)$$

where $\:{{\upbeta\:}}_{\mathrm{i}}$is a perturbation coefficient used to control the influence of the historical improvement direction. $\:{r}_{i}$ is a random number uniformly distributed in [0,1].The perturbation coefficient $\:{{\upbeta\:}}_{\mathrm{i}}$ is designed as a stochastic and iteration-dependent coefficient rather than a fixed empirical value. The upper bound of $\:{{\upbeta\:}}_{\mathrm{i}}$ is controlled by $\:1-\frac{\mathrm{t}}{\mathrm{T}}$. In the early evolutionary stage, $\:1-\frac{\mathrm{t}}{\mathrm{T}}$ is relatively large, allowing stronger perturbation along the historical improvement direction and enhancing exploration. As the iteration proceeds, $\:{{\upbeta\:}}_{\mathrm{i}}$ gradually decreases, which weakens unnecessary randomness and promotes stable convergence in the later stage.

17 $$\:{I}_{m}=\frac{{f}_{m}\left({pbest}_{i}\right)-{f}_{m}\left({x}_{i}\right)}{{f}_{m}^{max}-{f}_{m}^{min}+\epsilon\:},m=\mathrm{1,2}\cdot\:\cdot\:\cdot\:,M$$

where $\:\epsilon\:\:$is a small positive constant introduced to prevent zero division.

For minimization problems, $\:{I}_{m}>0$ indicates that the current position $\:{X}_{i}$ improves upon the previous personal best $\:{pbest}_{i}$ on the *m-th* objective; $\:{I}_{m}=0$ indicates no significant difference; and $\:{I}_{m}<0$ indicates deterioration on the corresponding objective. Compared with the previous discrete indicator, this continuous normalized indicator preserves the improvement magnitude of each objective.

18 $$\:{\lambda\:}_{i}=\frac{1}{2}\left[1+tanh\left(\frac{1}{{\rm\:M}}\sum\:_{m=1}^{M}{I}_{m}\right)\right]$$

Based on the improvement indicator $\:{I}_{m}$, the contribution weight $\:{\lambda\:}_{i}$ of the current position is calculated by Eq. (18). Since the hyperbolic tangent function maps the overall improvement degree into a bounded interval, $\:{\lambda\:}_{i}$ lies in (0,1). A larger $\:{\lambda\:}_{i}$ indicates that $\:{\mathrm{\:newposition}}_{i}$ provides stronger improvement over $\:{\mathrm{pbest}}_{i}^{\:prev}$ and should contribute more to the updated $\:\mathrm{pbest}$. Conversely, a smaller $\:{\lambda\:}_{i}$ means that the improvement is weak or unstable, and more historical information from $\:{\mathrm{c}}_{\mathrm{j}}\mathrm{best}$ should be retained.After the perturbed personal best is generated, its objective values are re-evaluated before it is used in subsequent dominance comparison and velocity update.

19 $$\:{\mathrm{pbest}}_{i}^{\:t+1}=clip({\mathrm{pbest}}_{i}^{\:prev}+{\xi\:}_{i},lb,ub)$$

$${\xi\:}_{i,d}\sim\:N(0,{\sigma\:}_{d}^{2}),{\sigma\:}_{d}=0.01({ub}_{d}-{lb}_{d}),\:\:d=\mathrm{1,2},\cdot\:\cdot\:\cdot\:,D$$

If ${\mathrm{\:newposition}}_{i}$ is dominated by $\:{\mathrm{pbest}}_{i}^{\:prev}$, or if neither solution dominates the other, and $\:{A}_{aux}$ is empty, Gaussian perturbation is used as a fallback update strategy. In Eq. (19), $\:{\xi\:}_{i}$ denotes a Gaussian perturbation vector sampled within the decision space, and clip(.) is used to keep the updated $\:\mathrm{pbest}$ inside the feasible decision domain.

20 $$\begin{aligned} pbest_{i}^{{t + 1}} & = clip(pbest_{i}^{{prev}} + r_{1} (c_{j} best - pbest_{i}^{{prev}} ) \\ & + r_{2} (c_{j} best - \:newposition_{i} ) + \xi _{i} ,lb,ub) \\ \end{aligned}$$

$$\:{r}_{1},{r}_{2}\sim\:\cup\:\left(\mathrm{0,1}\right)$$

When $\:{A}_{aux}$ is not empty, a historical solution $\:{\mathrm{c}}_{\mathrm{j}}\mathrm{best}$ is randomly selected from $\:{A}_{aux}$ to guide the $\:\mathrm{pbest}$ update. As shown in Eq. (20), the term $\:{\mathrm{c}}_{\mathrm{j}}\mathrm{best}$ - $\:{\mathrm{pbest}}_{i}^{\:prev}$ guides the personal best toward a historical high-quality region, while $\:{\mathrm{c}}_{\mathrm{j}}\mathrm{best}$-$\:{\mathrm{newposition}}_{i}$ introduces an additional correction direction to prevent the updated $\:\mathrm{pbest}$ from being overly affected by the current ineffective position. The Gaussian perturbation $\:{\xi\:}_{i}$ further enhances local exploration, and the clip(.) operation guarantees feasibility.

**Fig. 5:**
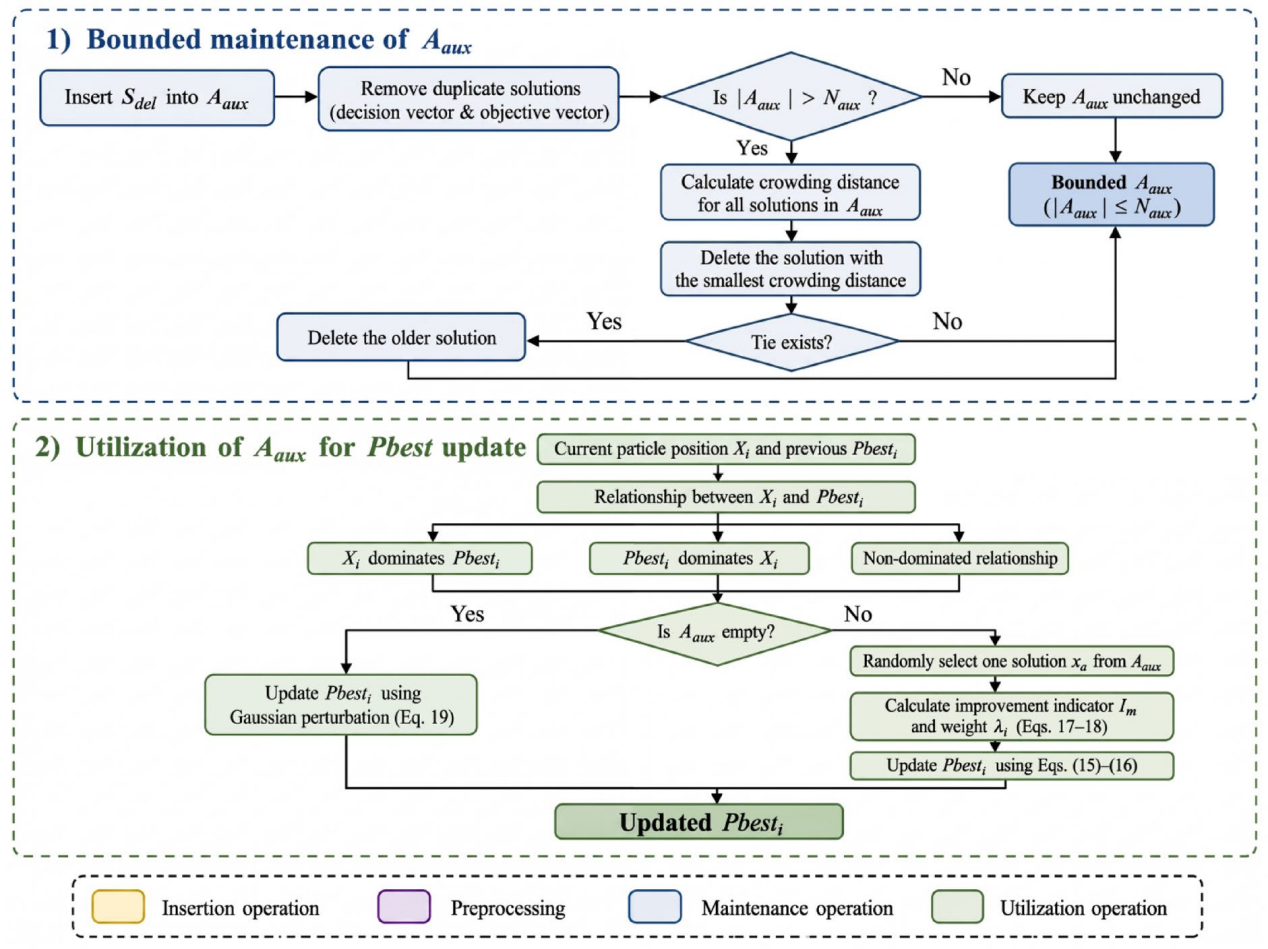
Flowchart of the bounded auxiliary archive maintenance and utilization process.

Figure 5 shows the flowchart of the bounded auxiliary archive maintenance and utilization process. The upper part describes how the auxiliary archive $\:{A}_{aux}$ is updated, including the insertion of $\:{S}_{del}$, duplicate removal, capacity checking, and crowding-distance-based maintenance. The lower part shows how $\:{A}_{aux}$ is used to assist the $\:\mathrm{pbest}$ update under different dominance relationships. This process ensures that the auxiliary archive remains bounded while providing historical guidance for particle learning.The specific implementation of this section’s strategy is detailed in Algorithm 3.

**Algorithm 3:**
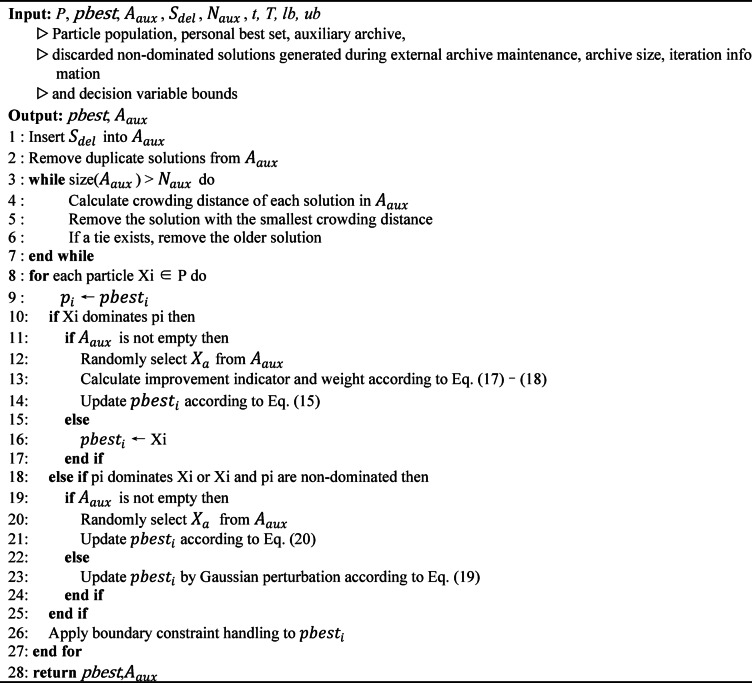
Pbest update based on bounded auxiliary archive.

### Particle reconstruction strategy based on stagnation detection

In MOPSO, particles may easily fall into local optimal regions or show weak learning ability. If such particles persistently adjust their flight trajectories based on their current positions, moving towards either their individual historical optimal solutions or the global optimum, effective improvement often proves elusive. A hallmark of this behavior is sustained stagnation across multiple consecutive iterations. This study defines the criterion for determining ‘stagnation’ as follows: if a particle’s new position fails to surpass its predecessor’s position, it is deemed stagnant.To accurately identify the particles requiring reconstruction, this study constructs a flight stagnation detector as follows:

21 $$s_{i} \left( {t + 1} \right) = \left\{ {\begin{array}{*{20}c} {0,} & {if\,x_{i} \left( {t + 1} \right) \prec x_{i} \left( t \right)} \\ {s_{i} \left( t \right) + 1,} & {otherwise} \\ \end{array} } \right.$$

When a particle is in an evolutionary state, that is, its new position supersedes its previous position, the detector count is reset to zero; otherwise, it is incremented by one. During each iteration, if a particle’s stagnation count reaches three, indicating three consecutive failures to evolve and a strong possibility of falling into a local optimum, the reconstruction mechanism should be triggered. The reconstruction threshold is set to three consecutive non-improving iterations to reduce false triggering caused by occasional random fluctuations. A single non-improving iteration does not necessarily indicate true stagnation, while an excessively large threshold may delay the reconstruction of ineffective particles and reduce the search efficiency. Particle reconstruction is not a simple random initialization. As random reinitialization may slow down algorithmic convergence and particles stored in the external archive typically lie closer to the Pareto front, the reconstruction strategy proposed herein combines particles from the external archive with the current particle information. This approach ensures that guided particles retain the excellent convergence properties of approximating the PF while introducing novel search directions for particles awaiting reconstruction, thereby effectively mitigating solution homogenization.

To prevent the reconstructed particles from becoming overly concentrated in localized regions of the Pareto front while maintaining the distribution diversity of the solution set, a random sampling mechanism based on crowding distance is employed when selecting guiding samples from the external archive. The specific procedure is as follows: as follows: first, the crowding-distance value of each archive particle is computed; a larger distance indicates a sparser solution distribution in the vicinity of that particle. Subsequently, the archive particles are sorted by crowding distance in descending order, selecting the top 30% of “high-diversity particles” to form the “candidate guiding set”. The reconstruction method is described below.

22 $$x_{i}^{{new}} \left( t \right) = \frac{{x_{i} \left( t \right) + sbest\left( {rand_{1} } \right) + sbest\left( {rand_{2} } \right)}}{3}$$

$sbest\left( {rand_{i} } \right)$ represents a random particle within the candidate guidance set. Upon completion of particle reconstruction, the corresponding stagnation detector count shall be reset to zero. Furthermore, it is necessary to verify whether the reconstructed particle lies within the boundary range of the decision variable. Should it exceed the boundary, correction shall be applied as follows:

23 $$x_{i} = \left\{ {\begin{array}{*{20}c} {ub_{i} } & {x^{\prime}_{i}> ub_{i} } \\ {lb_{i} } & {x^{\prime}_{i} < lb_{i} } \\ {x^{\prime}_{i} } & {lb_{i} \le ~x^{\prime}_{i} \le ub_{i} } \\ \end{array} } \right.$$

Where$\:\:\mathrm{u}\mathrm{b}$ and $\:\mathrm{l}\mathrm{b}$ denote the upper and lower bounds of the decision space in the i-th dimension respectively.

**Fig. 6:**
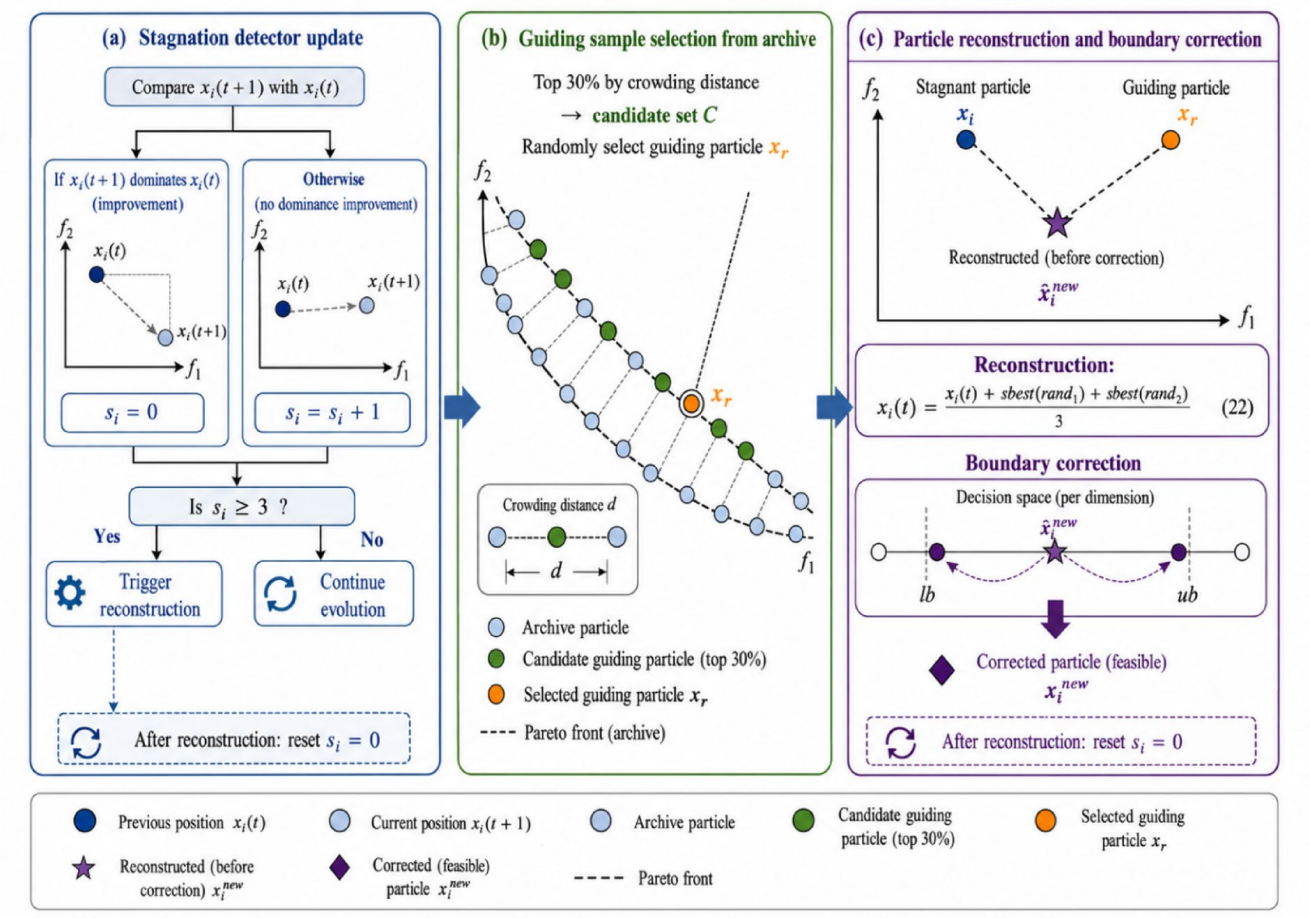
Schematic diagram of the particle reconstruction strategy.

Figure 6 illustrates the particle reconstruction strategy based on stagnation detection. First, the dominance status between the current particle position and the new position is examined to update the stagnation counter. If the particle fails to improve for three consecutive iterations, the reconstruction mechanism is triggered. Then, the archive particles are ranked based on crowding-distance values, and the top 30% sparsely distributed solutions are used to form the candidate guidance set. Finally, the stagnant particle is reconstructed using selected archive-guided samples, followed by boundary correction to ensure feasibility. This strategy helps stagnant particles return to promising search regions while maintaining population diversity.

Compared with traditional single-generation comparison-based decision mechanisms, the multi-generation cumulative stagnation detection mechanism proposed herein effectively avoids misjudgements caused by random perturbations by setting a threshold of “three consecutive generations without evolution”. This enables the precise identification of particles genuinely trapped in local optima, reducing unnecessary reconstruction operations, and thereby enhancing the overall computational efficiency of the algorithm.

This strategy employs Pareto-optimal particles from an external archive as reconstruction-guidance samples. Combined with an adaptive weighted fusion approach, it enables the reconstructed particles to approach the Pareto front more efficiently while retaining their original search experience. Algorithm 4 presents the detailed implementation procedure of this strategy.

**Algorithm 4:**
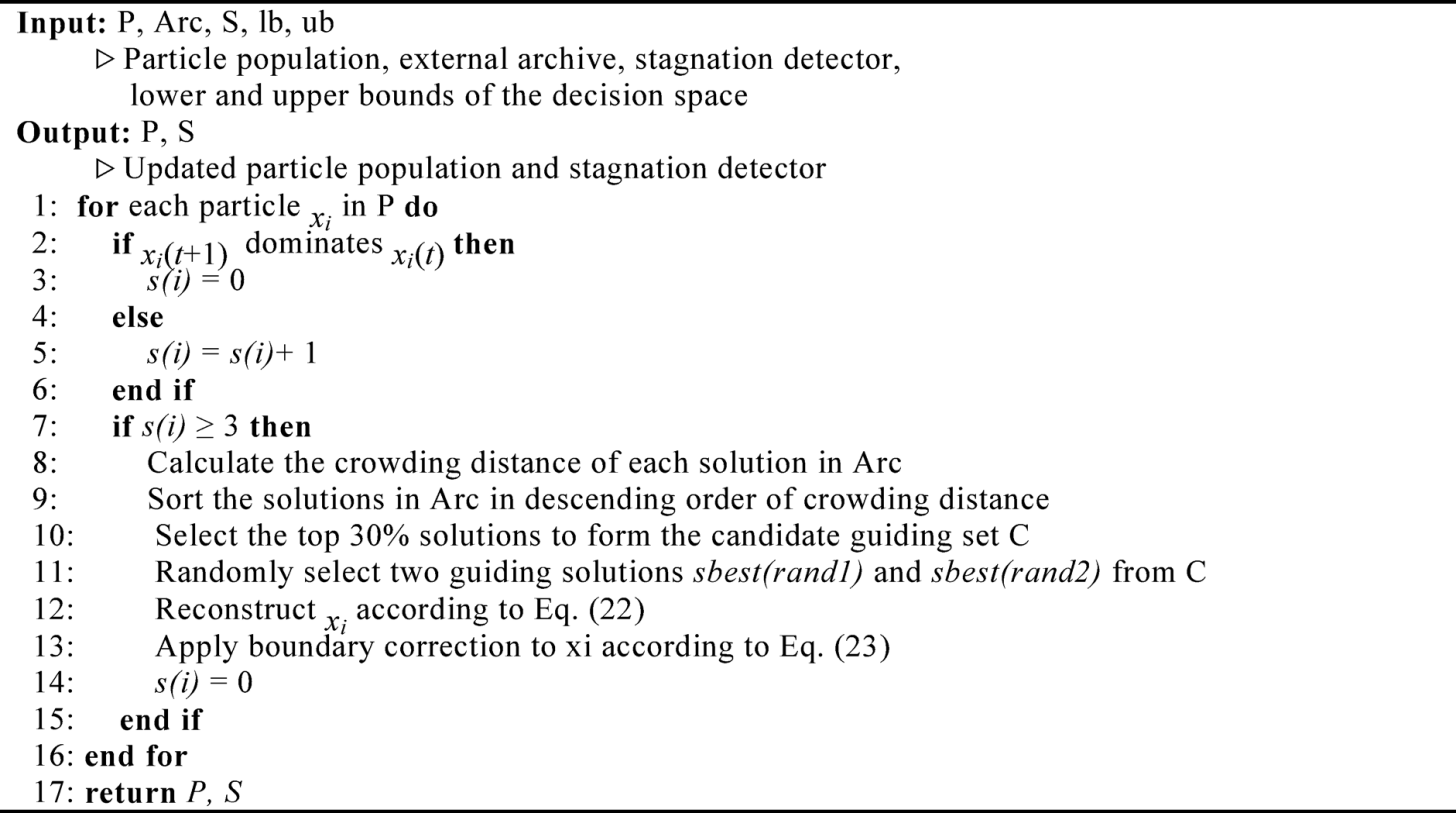
Particle reconstruction strategy based on stagnation detection.

## Experimental results and analysis

### Test problems and comparison algorithm

To evaluate the overall performance of the proposed TAMOPSO algorithm, three widely used benchmark suites, namely ZDT^38^, UF^39^, and DTLZ^40^, were adopted.The experimental setup included five bi-objective ZDT problems, seven bi-objective UF problems, three tri-objective UF problems, and seven tri-objective DTLZ problems. These benchmark problems have diverse levels of complexity and Pareto-front shapes, thereby providing a comprehensive assessment of the algorithm in terms of population diversity maintenance and convergence accuracy.

To provide a comprehensive evaluation, TAMOPSO was compared with five representative MOPSO algorithms, namely CMOPSO, MOPSOCD, MOPSO, MPSOD, and NMPSO. In addition, five advanced multi-objective evolutionary algorithms, namely AdaW, ARMOEA, CAMOEA, DGEA, and IDBEA, were included for further comparison. These benchmark algorithms have demonstrated outstanding competitiveness in multi-objective optimization and provide reliable reference points for performance evaluation.

### Algorithm performance indicators and parameter settings

The performance of the algorithms was assessed using two commonly used indicators: Inverted Generational Distance (IGD)^41^ and Hypervolume (HV)^42^. Together, these indicators provide an overall assessment of the convergence and diversity of the generated solution sets.

IGD calculates the mean of the shortest distances from reference points uniformly sampled on the true Pareto front (P∗) to the set of approximate solutions (*S*) obtained by the algorithm. Therefore, IGD can reflect both convergence and diversity, and its formulation is given below:

24 $$IGD(S,P^{*} ) = \frac{1}{{\left| {P^{*} } \right|}}\sum\limits_{{v \in P^{*} }} {\min _{{x \in s}} d(v,f(x))}$$

Here, *v* denotes a reference point in *p*∗, and |*p*∗| denotes the number of reference points. *f* (*x*) denotes the objective vector associated with a solution*\:x*in the approximate set S. *d*(*v*,*f* (*x*)) gives the Euclidean distance from the reference vector *v* tothe objective vector *f* (*x*). A small IGD value indicates that for every point on the true front, there exists a sufficiently close point in the approximate solution set.This signifies that the approximate solution set performs well in both convergence (proximity to the true front) and diversity (coverage of the entire front).

HV quantifies the objective-space volume dominated by the approximate solution set S with respect to a predefined reference point *r*. It provides an integrated assessment of convergence and diversity. Its formulation is given as:

25 $$HV(S,r) = \delta (U_{{X \in S}} [f_{1} (x),r_{1} ] \times ... \times [f_{m} (x),r_{m} ]$$

*r* = (*r*1, *r*2,…, *rm*) represents a reference point dominated by all solutions, where *m* denotes the number of objective functions and δ represents the Lebesgue measure. A higher HV value suggests that the approximate solution set covers a larger volume, This usually implies better convergence toward the true Pareto front and a wider, more uniform distribution in the objective space.

In this study, TAMOPSO was systematically compared with the ten algorithms mentioned above.For a fair comparison, the parameter settings of all compared algorithms followed those reported in the corresponding original studies, as listed in Table 1. Key parameter configurations for the remaining ten comparison algorithms across three test datasets—including the number of particles N, the number of objectives M, the dimensionality of the decision space D, and the maximum number of function evaluations FEs—are presented in Table 1. For each test problem, every algorithm was executed independently 30 times. All experiments were performed on a computer running Windows 11 and equipped with an AMD Ryzen 7 8845 H processor (integrated Radeon 780 M GPU, base frequency 3.80 GHz) using the MATLAB R2023b platform. The source code for the comparison algorithms was provided by the PlatEMO platform^43^.

**Table 1:** Parameter settings for the test functions.

Test functions	*N*	M	D	FEs
ZDT1−3	200	2	30	10,000
ZDT4, ZDT6	200	2	10	10,000
UF1−7	200	2	30	10,000
UF8−10	200	3	30	10,000
DTLZ1	200	3	7	10,000
DTLZ2−6	200	3	12	10,000
DTLZ7	200	3	22	10,000

Table 1 reports the common experimental configurations, including the number of particles N, the number of objectives M, the decision-space dimensionality D, and the maximum number of function evaluations (FEs). Algorithm-specific control parameters are given in Table 2.

$\:{\text{}\mathrm{p}}_{\mathrm{c}}$ and $\:{\text{}\mathrm{p}}_{m}$ represent the probabilities of crossover and mutation, respectively, while$\:{\text{}\eta}_{\mathrm{c}}$ and $\:{\text{}\eta}_{m}$ denote indicate the corresponding distribution indices.Other unspecified parameters followed the default settings in PlatEMO.

**Table 2:** Algorithms parameter configurations.

Algorithm	Parameter settings
CMOPSO	$\:{R}_{1},{R}_{1}\in\:\left[\mathrm{0,1}\right],r=10$
MOPSOCD	$\omega = 0.4,R_{1} ,R_{1} \in [0,1]$
MOPSO	$\omega = 0.4,c_{1} ,c_{2} , = 2$
MPSOD	$\omega \in\mathrm{[0.1,0.}\mathrm{9}\mathrm{]}\mathrm{,}\text{}{\mathrm{c}}_{\mathrm{1}}\mathrm{,}{\mathrm{c}}_{\mathrm{2}}\mathrm{=2}\mathrm{,}{\text{}\mathrm{p}}_{\mathrm{m}}\mathrm{=}\mathrm{0.1}\mathrm{,}\text{}{\eta}_{m}\mathrm{=20}\mathrm{,}{\text{}\eta}_{\mathrm{m}}\mathrm{=20}\mathrm{,\:F=0.5,CR=0.5}$
NMPSO	$\omega \in [0.1,0.5],c_{1} ,c_{2} ,c_{3} \in [1.5,2.5],p_{m} = 1/D,\eta _{m} = 20$
AdaW	$\:{{\text{}\mathrm{p}}_{\mathrm{c}}\mathrm{=}\mathrm{1.0,}\text{}\mathrm{p}}_{\mathrm{m}}\mathrm{=}\mathrm{1}\mathrm{/}\mathrm{D}\mathrm{,\:}{\eta}_{\mathrm{m}}\mathrm{=20}\mathrm{,}{\text{}\eta}_{\mathrm{c}}\mathrm{=20}$
ARMOEA	$\:{{\text{}\mathrm{p}}_{\mathrm{c}}\mathrm{=}\mathrm{1.0,}\text{}\mathrm{p}}_{\mathrm{m}}\mathrm{=}\mathrm{1}\mathrm{/}\mathrm{D}\mathrm{,\:}{\eta}_{\mathrm{m}}\mathrm{=20}\mathrm{,}{\text{}\eta}_{\mathrm{c}}\mathrm{=20}$
CAMOEA	$\:{{\text{}\mathrm{p}}_{\mathrm{c}}\mathrm{=}\mathrm{1.0,}\text{}\mathrm{p}}_{\mathrm{m}}\mathrm{=}\mathrm{1}\mathrm{/}\mathrm{D}\mathrm{,\:}{\eta}_{\mathrm{m}}\mathrm{=20}\mathrm{,}{\text{}\eta}_{\mathrm{c}}\mathrm{=20}$
DGEA	*\:R=10*
IDBEA	$\:{\text{}\mathrm{p}}_{\mathrm{c}}=1.0,{\text{}\mathrm{p}}_{\mathrm{m}}=1/\mathrm{D}$
TAMOPSO	$\omega = 0.4,c_{1} ,c_{2} , = 2,N_{{aux}} = 200,numgrid = 50,ep = 0.05 \cdot (1 + \frac{t}{T}) \cdot \sigma _{f}$

### Computational complexity analysis

Let N, A,M, and D represent the population size, the external archive capacity, the number of objectives, and the dimensionality of decision variables, respectively.$\:{C}_{f}$represents the computational expense required for a single objective-function evaluation. In the baseline MOPSO, the main computational operations in each iteration include objective-function evaluation, velocity and position update, personal-best update, leader selection from the external archive, and archive update.

The computational costs of objective-function evaluation, velocity-position updating, and dominance-based personal-best updating are** O(N**$\:{\boldsymbol{C}}_{\boldsymbol{f}}$**),O(ND),O(NM)**, respectively. Let K = A+N represent the total number of candidate solutions considered during archive updating.For baseline MOPSO, non-dominated sorting and archive maintenance usually have a worst-case complexity of **O(**${\boldsymbol{K}}^{2}\boldsymbol{M)}$. Accordingly, the time complexity of baseline MOPSO in each iteration can be estimated as **O(N**$\:{\mathbf{C}}_{\mathbf{f}}$**+ND+NM+**$\:{\mathbf{K}}^{2}\mathbf{M}$**)**.

Compared with baseline MOPSO, the proposed TAMOPSO retains the basic particle update framework but introduces three additional mechanisms: two-stage archive maintenance, bounded auxiliary archive-assisted *\:pbest* update, and stagnation-detection-based particle reconstruction. For the two-stage archive maintenance strategy, adaptive grid construction and high-density grid identification require **O(AM)**. After the high-density grid is identified, only the solutions located in this grid are further evaluated. Let S represent the number of candidate solutions contained in the identified high-density grid. The angle-based diversity evaluation requires pairwise angle calculation among these solutions, with a time complexity of **O(**$\:{\mathbf{S}}^{2}\mathbf{M}$**)**. The dual-distance convergence calculation requires **O(SM)**. Thus, the additional computational cost introduced by the two-stage archive maintenance strategy is **O(AM+**$\:{\mathbf{S}}^{2}\mathbf{M}$**+SM)**.

Since S refers only to the candidate solutions located in the identified high-density grid rather than all solutions in the external archive, S is usually much smaller than A. Therefore, this strategy avoids performing pairwise angle calculations over all archived solutions, and its additional cost remains controllable. For the bounded auxiliary archive, the discarded non-dominated solutions generated during external archive maintenance are inserted into $\:{\mathrm{A}}_{\mathrm{a}\mathrm{u}\mathrm{x}}$. Since the maximum size of $\:{\mathrm{A}}_{\mathrm{a}\mathrm{u}\mathrm{x}}$is bounded by N, the auxiliary archive does not grow without restriction. The additional computational cost mainly comes from inserting discarded solutions, removing duplicate solutions, and deleting crowded solutions when the auxiliary archive exceeds its capacity. The auxiliary archive-assisted *\:pbest* update is performed particle by particle, and its time complexity is **O(ND + NM)**. Regarding the particle reconstruction strategy based on stagnation detection, updating the stagnation counter requires **O(N)**. Particle reconstruction is triggered only when the stagnation counter reaches the predefined threshold. When reconstruction is triggered, the additional cost mainly comes from selecting sparse elite solutions from the external archive and reconstructing stagnant particles in the decision space. Therefore, compared with baseline MOPSO, the time complexity of TAMOPSO in each iteration can be formulated as **O(N**$\:{\mathbf{C}}_{\mathbf{f}}$**+ND+NM+**$\:{\mathbf{K}}^{2}\mathbf{M}$**) + O(AM+**$\:{\mathbf{S}}^{2}\mathbf{M}$ **+ SM+ND+NM)**. The first term corresponds to the basic MOPSO framework, while the second term corresponds to the additional mechanisms introduced by TAMOPSO. In the worst case, when S approaches A, the angle calculation may reach **O(**$\:{\boldsymbol{A}}^{2}\boldsymbol{M}$**)**.

However, in practical optimization, the angle and convergence evaluations are only performed in the selected high-density grid, so S is generally much smaller than the total archive size A. Thus, the additional computational cost of TAMOPSO is controllable.In terms of space complexity, baseline MOPSO mainly stores the particle population, velocity vectors, personal-best records, and external archive. Its space complexity can be expressed as **O(ND + AD)**. TAMOPSO additionally stores the bounded auxiliary archive and stagnation counters. Since the auxiliary archive capacity is limited to N, the extra storage requirement is **O(ND + NM)**. Accordingly, the total space complexity of TAMOPSO can be written as **O(ND + AD+ND + NM)**.

Since both the external archive and the auxiliary archive are bounded, TAMOPSO only moderately increases memory consumption compared with baseline MOPSO.

Overall, TAMOPSO introduces additional computational costs mainly from dense-grid evaluation, auxiliary archive maintenance, and stagnation-detection-based particle reconstruction. However, these additional operations are bounded by the archive size and are activated only under specific archive maintenance or stagnation conditions. In return, TAMOPSO improves archive quality, strengthens *\:pbest* guidance, preserves population diversity and improves the algorithm’s capability to escape local optima. Therefore, compared with baseline MOPSO, TAMOPSO trades a moderate increase in computational cost for improved solution quality.

## Experimental performance and result analysis

### Comparison with MOPSO

The comparative IGD and HV results are reported in Tables 3 and 4, where the best mean values are marked in bold.

Regarding the IGD metric, Table 3 indicates that TAMOPSO achieved the lowest IGD values in 11 of the 22 test problems.For ZDT1-ZDT3, TAMOPSO achieved the lowest IGD values with very small standard deviations, indicating outstanding performance in both convergence accuracy and operational stability.When addressing the multimodal problem ZDT4, its average IGD value surpasses that of the next-best CMOPSO algorithm by an order of magnitude. Despite the greater challenge posed by UF series problems, TAMOPSO achieved optimal performance on multiple problems. However, its performance fell short of optimal on the DTLZ2 and DTLZ4 problems, indicating that further improvement is still needed for certain complex three-objective problems.

Regarding the HV metric (Table 4), TAMOPSO produced the best HV results for 10 of the 22 test problems, significantly outperforming the other five multi-objective particle swarm comparison algorithms. In classic test problems, such as ZDT1 and ZDT3, TAMOPSO’s HV values consistently outperformed its competitors, such as CMOPSO. For the ZDT4 problem, TAMOPSO was the only algorithm that yielded a non-zero HV value, with all others recording zero values, demonstrating its exceptional ability to escape local optima. Furthermore, for challenging test problems such as DTLZ7, TAMOPSO maintained the capacity to produce valid HV values even when other algorithms failed entirely, demonstrating its robustness in complex environments.

**Table 3:** Comparison of IGD values for TAMOPSO and five MOPSO algorithms across 22 test problems.

Problem	M	D		CMOPSO	MOPSOCD	MOPSO	MPSOD	NMPSO	TAMOPSO
ZDT1	2	30	Mean Std	3.0265e-3 (3.41e-4)-	2.8370e-2 (5.78e-2)$\:\approx\:$	1.5422e + 0 (4.91e-2)-	1.0074e-1 (3.90e-2)-	3.6031e-2 (1.42e-2)-	**2.6717e-3** **(1.13e-4)**
ZDT2	2	30	Mean Std	2.8391e-3 (4.09e-4)$\:\approx\:$	1.4019e-1 (2.41e-1)-	3.0685e + 0 (1.39e-1)-	1.2678e-1 (7.59e-2)-	5.2607e-2 (1. 11e-1)-	**2.8110e-3** **(1.91e-4)**
ZDT3	2	30	Mean Std	3.5708e-3 (5.94e-4)-	4.5359e-2 (6.04e-2)-	1. 1622e + 0 (9.04e-2)-	1.9796e-1 (6.40e-2)-	9.3229e-2 (1.91e-2)-	**2.9266e-3** **(1.76e-4)**
ZDT4	2	10	Mean Std	2. 1535e + 1 (6.76e + 0)-	1.8376e + 1 (8.43e + 0)-	1.0430e + 1 (4.03e + 0)-	3.4853e + 1 (7.25e + 0)-	1.7842e + 1 (1.27e + 1)-	**7.4174e-1** **(2.54e-1)**
ZDT6	2	10	Mean Std	**1.5709e-3** **(3.17e-5)+**	3.7392e-3 (2.84e-3)-	5.5419e + 0 (3.45e-1)-	2.6476e-2 (5. 16e-2)-	2.3778e-3 (3.80e-4)$\:\approx\:$	2.2849e-3 (2.72e-4)
UF1	2	30	Mean Std	**1.0015e-1** **(2.39e-2)+**	6.9328e-1 (1.26e-1)-	4.6361e-1 (6.89e-2)-	2.6903e-1 (4. 10e-2)-	1.2105e-1 (1.53e-2)+	1.4091e-1 (1. 15e-2)
UF2	2	30	Mean Std	**6.1626e-2** **(5.59e-3)+**	1.3790e-1 (1.52e-2)-	1.0420e-1 (9.87e-3)-	1. 1670e-1 (1.06e-2)-	8.3052e-2 (5.56e-3)-	7.4648e-2 (3.85e-3)
UF3	2	30	Mean Std	3.9102e-1 (3.74e-2)-	3.8957e-1 (8.87e-2)-	4.5224e-1 (2.72e-2)-	5.0585e-1 (1.45e-2)-	3.5789e-1 (5.55e-2)-	**3.1356e-1** **(3.75e-2)**
UF4	2	30	Mean Std	1. 1163e-1 (8. 12e-3)-	7.7435e-2 (9.46e-3)-	1.8261e-1 (4.02e-3)-	9.8268e-2 (5.85e-3)-	**6.1501e-2** **(7.34e-3)+**	6.3882e-2 (5.93e-3)
UF5	2	30	Mean Std	**9.6848e-1** **(2.17e-1)+**	3.8937e + 0 (4.37e-1)-	2.5722e + 0 (3.49e-1)-	2.6794e + 0 (2.48e-1)-	1.6538e + 0 (4.38e-1)$\:\approx\:$	1.6064e + 0 (2.26e-1)
UF6	2	30	Mean Std	**3.9182e-1** **(6.77e-2)+**	2.8060e + 0 (5. 16e-1)-	2. 1540e + 0 (3.76e-1)-	1.2739e + 0 (1.80e-1)-	6.7698e-1 (1.44e-1)$\:\approx\:$	7.0799e-1 (9.33e-2)
UF7	2	30	Mean Std	1.2142e-1 (1.09e-1)$\:\approx\:$	6.2651e-1 (1. 11e-1)-	5.7874e-1 (8.00e-2)-	2.5445e-1 (7.61e-2)-	2.3075e-1 (2.03e-1)-	**1.0760e-1** **(1.47e-2)**
UF8	3	30	Mean Std	5.9784e-1 (1.06e-1)-	7.4377e-1 (1.50e-1)-	4.3139e-1 (2.80e-2)-	5.3082e-1 (3.06e-2)-	4.7212e-1 (5. 10e-2)-	**2.8518e-1** **(1.01e-1)**
UF9	3	30	Mean Std	8.9549e-1 (1.37e-1)-	8.9512e-1 (1.44e-1)-	5.4313e-1 (4.50e-2)-	6.5561e-1 (3.94e-2)-	4.5986e-1 (6.74e-2)-	**1.3723e-1** **(1.19e-2)**
UF10	3	30	Mean Std	4.3262e + 0 (4.21e-1)-	5.0499e + 0 (8.49e-1)-	1.4436e + 0 (2.25e-1)-	4.2243e + 0 (2.87e-1)-	1.4973e + 0 (3.88e-1)-	**8.6257e-1** **(1.89e-1)**
DTLZ1	3	7	Mean Std	1.5182e + 1 (3.66e + 0)$\:\approx\:$	1.7791e + 1 (6.27e + 0)-	**5.1809e + 0** **(2.53e + 0)+**	1.0221e + 1 (2.66e + 0)+	6.0516e + 0 (3.36e + 0)-	1.4513e + 1 (4.97e + 0)
DTLZ2	3	12	Mean Std	**4.1464e-2** **(6.86e-4)+**	9.5136e-2 (9.05e-3)+	4.8275e-2 (2.32e-3)+	4.5623e-2 (1.42e-3)+	5.5887e-2 (1.76e-3)+	3.6070e-1 (6. 13e-2)
DTLZ3	3	12	Mean Std	1.9850e + 2 (4. 19e + 1)-	1.3279e + 2 (3.36e + 1)-	1.7761e + 2 (4.79e + 1)-	1.5091e + 2 (1.72e + 1)-	1. 1530e + 2 (2.70e + 1)$\:\approx\:$	**1.0491e + 2** **(3.61e + 1)**
DTLZ4	3	12	Mean Std	**4.7674e-2** **(1.91e-2)+**	3.2486e-1 (4.68e-2)-	2.2166e-1 (1.22e-1)+	1.3379e-1 (4.09e-2)+	5.6932e-2 (1.72e-3)+	2.8791e-1 (3.26e-2)
DTLZ5	3	12	Mean Std	**4.6931e-3** **(3.07e-4)+**	3.6791e-2 (9.79e-3)-	4.8210e-3 (3.65e-4)+	5.6083e-2 (6.07e-3)-	7. 1061e-3 (1.00e-3)-	6. 1633e-3 (7.98e-4)
DTLZ6	3	12	Mean Std	1.4217e-1 (3.22e-1)$\:\approx\:$	9.2088e-2 (2.36e-1)-	7.4330e + 0 (2.70e-1)-	1.0863e + 0 (3.46e-1)-	1.3632e-2 (2.69e-3)-	**3.0288e-3** **(2.74e-4)**
DTLZ7	3	22	Mean Std	4.9504e-2 (2.70e-3)+	1.0975e-1 (1.26e-1)+	6.5366e + 0 (4.64e-1)-	5.4732e-1 (1.04e-1)-	**4.6655e-2** **(2.50e-3)+**	3.4423e-1 (1.26e-1)
Best/all				**8/22**	**0/22**	**1/22**	**0/22**	**2/22**	**11/22**
*- / + / ≈*				**9/9/4**	**19/2/1**	**18/4/0**	**19/3/0**	**13/5/4**	

**Table 4:** Comparison of HV values for TAMOPSO and five MOPSO algorithms across 22 test problems.

Problem	M	D		CMOPSO	MOPSOCD	MOPSO	MPSOD	NMPSO	TAMOPSO
ZDT1	2	30	Mean Std	7.2008e-1 (5.48e-4)-	6.8963e-1 (7.09e-2)-	0.0000e + 0 (0.00e + 0)-	5.7391e-1 (5.54e-2)-	6.8234e-1 (1.59e-2)-	**7.2141e-1** **(1.65e-4)**
ZDT2	2	30	Mean Std	4.4483e-1 (7.22e-4)-	3.4527e-1 (1.60e-1)-	0.0000e + 0 (0.00e + 0)-	2.9046e-1 (7.92e-2)-	4.0931e-1 (8.43e-2)-	**4.4596e-1** **(2.08e-4)**
ZDT3	2	30	Mean Std	6.0006e-1 (1.09e-3)$\:\approx\:$	5.7417e-1 (4.33e-2)-	1.4820e-3 (3. 14e-3)-	4.7301e-1 (5. 19e-2)-	5.7051e-1 (7.48e-3)-	**6.0026e-1** **(4.55e-4)**
ZDT4	2	10	Mean Std	0.0000e + 0 (0.00e + 0)-	0.0000e + 0 (0.00e + 0)-	0.0000e + 0 (0.00e + 0)-	0.0000e + 0 (0.00e + 0)-	0.0000e + 0 (0.00e + 0)-	**1.6632e-1** **(1.91e-1)**
ZDT6	2	10	Mean Std	**3.9035e-1** **(3.91e-5)+**	3.8827e-1 (2.92e-3)-	0.0000e + 0 (0.00e + 0)-	3.7026e-1 (2.90e-2)-	3.8970e-1 (3.26e-4)$\:\approx\:$	3.8963e-1 (2.49e-4)
UF1	2	30	Mean Std	**5.7121e-1** **(1.80e-2)+**	5.0816e-2 (5.07e-2)+	1.8880e-1 (4.97e-2)-	3.5489e-1 (3.98e-2)-	5.3416e-1 (2.64e-2)+	5.0151e-1 (1.90e-2)
UF2	2	30	Mean Std	**6.4543e-1** **(6.20e-3)+**	5.4483e-1 (1.97e-2)-	6.0862e-1 (7.03e-3)-	5.7552e-1 (1.41e-2)-	6. 1770e-1 (7. 18e-3)-	6.3497e-1 (3.67e-3)
UF3	2	30	Mean Std	2.6432e-1 (2.57e-2)-	2.4838e-1 (6.45e-2)-	1.8516e-1 (2. 18e-2)-	1.6772e-1 (1.21e-2)-	2.8806e-1 (4.23e-2)-	**3.4365e-1** **(3.54e-2)**
UF4	2	30	Mean Std	2.8983e-1 (9.94e-3)-	3.3445e-1 (1.28e-2)-	2. 1163e-1 (3.42e-3)-	3.0892e-1 (7.04e-3)-	**3.6256e-1** **(1.12e-2)+**	3.5457e-1 (7.49e-3)
UF5	2	30	Mean Std	**6.0392e-3** **(1.79e-2)+**	0.0000e + 0 (0.00e + 0)$\:\approx\:$	0.0000e + 0 (0.00e + 0)$\:\approx\:$	0.0000e + 0 (0.00e + 0)$\:\approx\:$	0.0000e + 0 (0.00e + 0)$\:\approx\:$	0.0000e + 0 (0.00e + 0)
UF6	2	30	Mean Std	**1.3867e-1** **(6.59e-2)+**	0.0000e + 0 (0.00e + 0)-	0.0000e + 0 (0.00e + 0)-	0.0000e + 0 (0.00e + 0)-	1.9826e-2 (3.05e-2)$\:\approx\:$	8.3190e-3 (1.03e-2)
UF7	2	30	Mean Std	**4.4650e-1** **(8.25e-2)** $\:\approx\:$	2. 1966e-2 (2.51e-2)-	6. 1610e-2 (3.91e-2)-	2.5730e-1 (6.94e-2)-	3.6073e-1 (1.30e-1)-	4.2245e-1 (2. 18e-2)
UF8	3	30	Mean Std	1.8000e-2 (2.23e-2)-	8.5876e-3 (1.44e-2)-	2.6407e-1 (2.08e-2)-	6. 1760e-2 (2.04e-2)-	2.9491e-1 (2.70e-2)-	**3.3999e-1** **(2.10e-2)**
UF9	3	30	Mean Std	2.4165e-2 (3.80e-2)-	2.5251e-2 (2.77e-2)-	2.5946e-1 (2.73e-2)-	1. 1202e-1 (2.73e-2)-	3. 1534e-1 (5.54e-2)-	**6.1990e-1** **(1.47e-2)**
UF10	3	30	Mean Std	0.0000e + 0 (0.00e + 0)-	0.0000e + 0 (0.00e + 0)-	0.0000e + 0 (0.00e + 0)-	0.0000e + 0 (0.00e + 0)-	0.0000e + 0 (0.00e + 0)-	**1.2291e-1** **(7.76e-2)**
DTLZ1	3	7	Mean Std	0.0000e + 0 (0.00e + 0)-	0.0000e + 0 (0.00e + 0)-	0.0000e + 0 (0.00e + 0)-	0.0000e + 0 (0.00e + 0)-	0.0000e + 0 (0.00e + 0)-	**5.5021e-3** **(2.96e-2)**
DTLZ2	3	12	Mean Std	5.5885e-1 (1.44e-3)+	4.7462e-1 (1.51e-2)+	5.4232e-1 (6.77e-3)+	5.4878e-1 (3.04e-3)+	**5.6930e-1** **(1.07e-3)+**	2.7373e-1 (3.46e-2)
DTLZ3	3	12	Mean Std	0.0000e + 0 (0.00e + 0)$\:\approx\:$	0.0000e + 0 (0.00e + 0)$\:\approx\:$	0.0000e + 0 (0.00e + 0)$\:\approx\:$	0.0000e + 0 (0.00e + 0)$\:\approx\:$	0.0000e + 0 (0.00e + 0)$\:\approx\:$	0.0000e + 0 (0.00e + 0)
DTLZ4	3	12	Mean Std	5.5478e-1 (8.62e-3)+	2.6981e-1 (6.32e-2)-	4.6116e-1 (5.26e-2)+	4.3212e-1 (4.93e-2)+	**5.6929e-1** **(1.08e-3)+**	3.5013e-1 (4.23e-2)
DTLZ5	3	12	Mean Std	**1.9880e-1** **(3.31e-4)+**	1.7122e-1 (9.80e-3)-	1.9848e-1 (9.61e-4)+	1.4053e-1 (8.43e-3)-	1.9804e-1 (6.76e-4)+	1.9710e-1 (6.77e-4)
DTLZ6	3	12	Mean Std	1.6879e-1 (7. 13e-2)$\:\approx\:$	1.8084e-1 (4.56e-2)-	0.0000e + 0 (0.00e + 0)-	3.9456e-3 (1.68e-2)-	1.9773e-1 (7.48e-4)-	**2.0066e-1** **(2.83e-4)**
DTLZ7	3	22	Mean Std	2.7252e-1 (2.42e-3)+	2.5576e-1 (4.77e-2)+	0.0000e + 0 (0.00e + 0)+	6.2174e-2 (3.48e-2)-	**2.8151e-1** **(8.70e-4)+**	8.8424e-2 (2.57e-2)
Best/all				**7/22**	**0/22**	**0/22**	**0/22**	**4/22**	**10/22**
*- / + / ≈*				**9/8/4**	**16/3/3**	**15/4/3**	**17/2/3**	**11/6/5**	

### Comparison with MOEAs

Tables 5 and 6 present the comparative results of TAMOPSO against five multi-objective evolutionary algorithms (MOEAs) on the HV and IGD metrics across 22 test problems, with optimal values highlighted in bold.

Regarding the IGD metric (Table 5), TAMOPSO demonstrated a more pronounced advantage than the other algorithms. Across 22 test problems, TAMOPSO achieved optimal IGD values in 12 instances. For the majority of ZDT and UF problems, the IGD values were one order of magnitude lower than those of the five evolutionary algorithms under comparison, or even more so. on UF3, UF4, UF7, and UF9, its IGD values were markedly lower than those of all the comparison algorithms, demonstrating its superiority in handling complex-front shapes. However, for more challenging multimodal problems, such as DTLZ3, TAMOPSO exhibited poorer IGD performance, indicating that further optimization is required when addressing high-dimensional complex target spaces.

Regarding the HV metric (Table 6), TAMOPSO achieved the best HV value on 10 of the 22 test problems, significantly outperforming the other comparison algorithms (the best-performing comparative algorithm, CMOPSO, led only seven problems). For the ZDT4 problem, which features numerous local optima, TAMOPSO was the only algorithm to achieve a non-zero mean value for HV, whereas all other comparative algorithms failed. Across the UF3, UF7, UF8, and UF9 test problems, the algorithm demonstrated optimal performance, particularly for UF9, where its HV value substantially exceeded those of the other algorithms. On the DTLZ1 problem, despite an unfavorable IGD value, the HV metric indicated TAMOPSO as the sole algorithm that yielded a non-zero solution set.

**Table 5:** Comparison of IGD values for TAMOPSO and five MOEAs algorithms across 22 test problems.

Problem	M	D		AdaW	ARMOEA	CAMOEA	DGEA	IDBEA	TAMOPSO
ZDT1	2	30	Mean Std	1.3819e-1 (1.94e-2)-	5.8811e-2 (1.94e-2)-	6.4892e-2 (8.53e-3)-	1.0701e + 0 (2.38e-1)-	5. 1957e-1 (6.61e-2)-	**2.6717e-3** **(1.13e-4)**
ZDT2	2	30	Mean Std	1.8065e-1 (3.03e-2)-	6.4481e-1 (1.37e-1)-	1.4158e-1 (1.00e-1)-	1.0235e + 0 (3.00e-1)-	1.9545e + 0 (3.25e-1)-	**2.8110e-3** **(1.91e-4)**
ZDT3	2	30	Mean Std	1. 1573e-1 (2.06e-2)-	5. 1379e-2 (1.53e-2)-	5.8056e-2 (9.61e-3)-	1.0026e + 0 (2.38e-1)-	3.3156e-1 (5. 10e-2)-	**2.9266e-3** **(1.76e-4)**
ZDT4	2	10	Mean Std	1.7969e + 0 (4.78e-1)-	1.4832e + 0 (5.02e-1)-	1.8658e + 0 (5.29e-1)-	7.5230e + 0 (5.28e + 0)-	2.8762e + 1 (6.39e + 0)-	**7.4174e-1** **(2.54e-1)**
ZDT6	2	10	Mean Std	9.4892e-1 (1.99e-1)-	8. 1137e-1 (2.01e-1)-	1. 1678e + 0 (1.94e-1)-	2.5549e-2 (7.77e-2)$\:\approx\:$	4.5353e + 0 (3.36e-1)-	**2.2849e-3** **(2.72e-4)**
UF1	2	30	Mean Std	1.4105e-1 (2.34e-2)$\:\approx\:$	1.2853e-1 (3.70e-2)$\:\approx\:$	**1.1168e-1** **(2.16e-2)+**	6.7105e-1 (1.48e-1)-	3.9407e-1 (7.43e-2)-	1.4091e-1 (1. 15e-2)
UF2	2	30	Mean Std	7.3797e-2 (1. 18e-2)$\:\approx\:$	**7.2612e-2** **(5.46e-3)+**	7.6307e-2 (4.58e-3)$\:\approx\:$	1.7197e-1 (2.49e-2)-	1.5884e-1 (1.84e-2)-	7.4648e-2 (3.85e-3)
UF3	2	30	Mean Std	4.3300e-1 (1.64e-2)-	4.2719e-1 (3. 19e-2)-	4.6259e-1 (1.63e-2)-	5.6367e-1 (4. 16e-2)-	6.0870e-1 (2.01e-2)-	**3.1356e-1** **(3.75e-2)**
UF4	2	30	Mean Std	8. 1951e-2 (3. 10e-3)-	8.2362e-2 (3.46e-3)-	8.7566e-2 (2.25e-3)-	1.2024e-1 (1.08e-2)-	1.0580e-1 (3.66e-3)-	**6.3882e-2** **(5.93e-3)**
UF5	2	30	Mean Std	1. 1717e + 0 (1.91e-1)+	**7.3549e-1** **(2.42e-1)+**	1.0714e + 0 (2.93e-1)+	2.9319e + 0 (5.79e-1)-	3.0583e + 0 (2.34e-1)-	1.6064e + 0 (2.26e-1)
UF6	2	30	Mean Std	6.9601e-1 (7.65e-2)$\:\approx\:$	**5.2122e-1** **(7.27e-2)+**	5.9975e-1 (1. 15e-1)+	2.6498e + 0 (6.79e-1)-	2.0043e + 0 (3.53e-1)-	7.0799e-1 (9.33e-2)
UF7	2	30	Mean Std	2.0484e-1 (7.93e-2)-	2.2898e-1 (1. 17e-1)-	1.7294e-1 (7.64e-2)-	7.2170e-1 (1.25e-1)-	4.4246e-1 (9. 11e-2)-	**1.0760e-1** **(1.47e-2)**
UF8	3	30	Mean Std	3. 1063e-1 (2.01e-2)$\:\approx\:$	3.5469e-1 (4.96e-2)-	3.3542e-1 (3.61e-2)-	7.0195e-1 (1.51e-1)-	3.6924e-1 (4. 19e-2)-	**2.8518e-1** **(1.01e-1)**
UF9	3	30	Mean Std	4.4384e-1 (7.53e-2)-	4.8482e-1 (5.91e-2)-	4.7745e-1 (4.54e-2)-	7.9968e-1 (1.21e-1)-	5.5242e-1 (5.90e-2)-	**1.3723e-1** **(1.19e-2)**
UF10	3	30	Mean Std	1.9710e + 0 (3.64e-1)-	1.0801e + 0 (3.03e-1)-	1.6261e + 0 (3.87e-1)-	4.6286e + 0 (1.01e + 0)-	3.2176e + 0 (4.47e-1)-	**8.6257e-1** **(1.89e-1)**
DTLZ1	3	7	Mean Std	8.4357e-1 (4.82e-1)+	**3.9489e-1** **(1.83e-1)+**	6.0283e-1 (3. 17e-1)+	1.3257e + 1 (7.68e + 0)$\:\approx\:$	2.7983e + 0 (8.80e-1)+	1.4513e + 1 (4.97e + 0)
DTLZ2	3	12	Mean Std	4. 1149e-2 (1. 11e-3)-	**3.9532e-2** **(5.15e-4)-**	4.2058e-2 (6.43e-4)-	1. 1514e-1 (1.69e-2)-	4.3358e-2 (1.57e-3)-	3.6070e-1 (6. 13e-2)
DTLZ3	3	12	Mean Std	2.5755e + 1 (8.94e + 0)+	**1.6609e + 1** **(6.66e + 0)+**	2.6145e + 1 (6.94e + 0)+	1.0200e + 2 (5.96e + 1)$\:\approx\:$	7.6968e + 1 (1.38e + 1)+	1.0491e + 2 (3.61e + 1)
DTLZ4	3	12	Mean Std	**4.1174e-2** **(9.57e-4)+**	1.0645e-1 (1.73e-1)+	4.3141e-2 (6.09e-4)+	4.3842e-1 (1.38e-1)-	7.8929e-2 (1.27e-1)+	2.8791e-1 (3.26e-2)
DTLZ5	3	12	Mean Std	6.6540e-3 (1.22e-3)$\:\approx\:$	**4.6842e-3** **(5.89e-4)+**	5.4826e-3 (5.40e-4)+	5.8114e-2 (1.27e-2)-	1.2087e-2 (1.64e-3)-	6. 1633e-3 (7.98e-4)
DTLZ6	3	12	Mean Std	2.4745e-2 (1.43e-2)-	9.6993e-3 (1.07e-2)-	2. 1117e-2 (1.41e-2)-	2. 1654e + 0 (9.53e-1)-	7.3036e-1 (3.38e-1)-	**3.0288e-3** **(2.74e-4)**
DTLZ7	3	22	Mean Std	1.7374e-1 (4.32e-2)+	2.2624e-1 (1.92e-1)+	**1.2146e-1** **(2.10e-2)+**	4.5340e + 0 (1.57e + 0)-	1. 1078e + 0 (1.23e + 0)-	3.4423e-1 (1.26e-1)
Best/all				**1/22**	**7/22**	**2/22**	**0/22**	**0/22**	**12/22**
*- / + / ≈*				**12/5/5**	**13/8/1**	**13/8/1**	**19/0/3**	**20/2/0**	

**Table 6:** Comparison of HV values for TAMOPSO and five MOEAs algorithms across 22 test problems.

Problem	M	D	Type	AdaW	ARMOEA	CAMOEA	DGEA	IDBEA	TAMOPSO
ZDT1	2	30	Mean Std	5.3881e-1 (2.48e-2)-	6.4919e-1 (1.57e-2)-	6.3565e-1 (1. 13e-2)-	1.3628e-2 (3.30e-2)-	1.5412e-1 (3.91e-2)-	**7.2141e-1** **(1.65e-4)**
ZDT2	2	30	Mean Std	2.0903e-1 (2.97e-2)-	1.8770e-2 (6.39e-2)-	2.7387e-1 (5.75e-2)-	5.9799e-3 (2.09e-2)-	0.0000e + 0 (0.00e + 0)-	**4.4596e-1** **(2.08e-4)**
ZDT3	2	30	Mean Std	5.2571e-1 (1.63e-2)-	5.8380e-1 (3.47e-2)$\:\approx\:$	5.6141e-1 (6.35e-3)-	3.4841e-2 (5.08e-2)-	3.8245e-1 (4.45e-2)-	**6.0026e-1** **(4.55e-4)**
ZDT4	2	10	Mean Std	0.0000e + 0 (0.00e + 0)-	2.2645e-3 (1.07e-2)-	1. 1693e-3 (6.40e-3)-	0.0000e + 0 (0.00e + 0)-	0.0000e + 0 (0.00e + 0)-	**1.6632e-1** **(1.91e-1)**
ZDT6	2	10	Mean Std	1.2655e-3 (6.93e-3)-	2.8447e-3 (7.83e-3)-	0.0000e + 0 (0.00e + 0)-	3.7653e-1 (4. 15e-2)$\:\approx\:$	0.0000e + 0 (0.00e + 0)-	**3.8963e-1** **(2.49e-4)**
UF1	2	30	Mean Std	5.0940e-1 (3.32e-2)$\:\approx\:$	**5.5471e-1** **(3.25e-2)+**	5.4745e-1 (3. 10e-2)+	7.7314e-2 (7.79e-2)-	2.3242e-1 (7.27e-2)-	5.0151e-1 (1.90e-2)
UF2	2	30	Mean Std	6.2908e-1 (9.89e-3)-	6.3311e-1 (5.55e-3)$\:\approx\:$	6.2693e-1 (4.52e-3)-	5. 1533e-1 (1.99e-2)-	5.0773e-1 (2.42e-2)-	**6.3497e-1** **(3.67e-3)**
UF3	2	30	Mean Std	2. 1201e-1 (1.63e-2)-	2.0915e-1 (2.63e-2)-	1.9651e-1 (1.25e-2)-	1.2332e-1 (2.42e-2)-	9.5820e-2 (1.29e-2)-	**3.4365e-1** **(3.54e-2)**
UF4	2	30	Mean Std	3.2858e-1 (3.80e-3)-	3.3233e-1 (3.94e-3)-	3.2427e-1 (3.29e-3)-	2.7945e-1 (1.26e-2)-	2.9756e-1 (4.27e-3)-	**3.5457e-1** **(7.49e-3)**
UF5	2	30	Mean Std	0.0000e + 0 (0.00e + 0)$\:\approx\:$	**2.4643e-2** **(3.80e-2)+**	1.3393e-3 (7.06e-3)$\:\approx\:$	0.0000e + 0 (0.00e + 0)$\:\approx\:$	0.0000e + 0 (0.00e + 0)$\:\approx\:$	0.0000e + 0 (0.00e + 0)
UF6	2	30	Mean Std	1.7453e-2 (1.92e-2)+	**4.6656e-2** **(2.63e-2)+**	2.9151e-2 (2.42e-2)+	0.0000e + 0 (0.00e + 0)-	0.0000e + 0 (0.00e + 0)-	8.3190e-3 (1.03e-2)
UF7	2	30	Mean Std	3.3987e-1 (5.88e-2)-	3.4990e-1 (7.71e-2)-	3.6518e-1 (6.71e-2)-	1.9982e-2 (3.36e-2)-	1.0541e-1 (5.99e-2)-	**4.2245e-1** **(2.18e-2)**
UF8	3	30	Mean Std	2.2596e-1 (3.28e-2)-	2.9813e-1 (2.79e-2)-	2.5789e-1 (4. 11e-2)-	3.0584e-2 (3.02e-2)-	1.5764e-1 (4.57e-2)-	**3.3999e-1** **(2.10e-2)**
UF9	3	30	Mean Std	3.0696e-1 (6. 15e-2)-	2.9748e-1 (3.96e-2)-	2.8371e-1 (3.58e-2)-	5.9852e-2 (5. 11e-2)-	2.0268e-1 (4.40e-2)-	**6.1990e-1** **(1.47e-2)**
UF10	3	30	Mean Std	0.0000e + 0 (0.00e + 0)-	0.0000e + 0 (0.00e + 0)-	0.0000e + 0 (0.00e + 0)-	0.0000e + 0 (0.00e + 0)-	0.0000e + 0 (0.00e + 0)-	**1.2291e-1** (7.76e-2)
DTLZ1	3	7	Mean Std	1.6194e-2 (4.74e-2)$\:\approx\:$	**1.3331e-1** **(2.14e-1)+**	5. 1644e-2 (1.09e-1)+	9.2649e-3 (5.07e-2)$\:\approx\:$	0.0000e + 0 (0.00e + 0)-	5.5021e-3 (2.96e-2)
DTLZ2	3	12	Mean Std	**5.6509e-1** **(1.55e-3)+**	5.6491e-1 (1.33e-3)+	5.5897e-1 (1.71e-3)+	4.2579e-1 (2.59e-2)+	5.5402e-1 (3.04e-3)+	2.7373e-1 (3.46e-2)
DTLZ3	3	12	Mean Std	0.0000e + 0 (0.00e + 0)$\:\approx\:$	0.0000e + 0 (0.00e + 0)$\:\approx\:$	0.0000e + 0 (0.00e + 0)$\:\approx\:$	0.0000e + 0 (0.00e + 0)$\:\approx\:$	0.0000e + 0 (0.00e + 0)$\:\approx\:$	0.0000e + 0 (0.00e + 0)
DTLZ4	3	12	Mean Std	**5.6536e-1** **(1.35e-3)+**	5.3618e-1 (7.54e-2)+	5.5937e-1 (1.48e-3)+	1.9438e-1 (9.01e-2)-	5.3623e-1 (5.73e-2)+	3.5013e-1 (4.23e-2)
DTLZ5	3	12	Mean Std	1.9711e-1 (2.82e-3)+	**1.9858e-1** **(4.82e-4)+**	1.9802e-1 (3.62e-4)+	1.4767e-1 (1.70e-2)-	1.9155e-1 (1.58e-3)-	1.9710e-1 (6.77e-4)
DTLZ6	3	12	Mean Std	1.8464e-1 (1.05e-2)-	1.9389e-1 (8.95e-3)-	1.8627e-1 (1.07e-2)-	7.6998e-3 (3.39e-2)-	1.0882e-2 (2. 12e-2)-	**2.0066e-1** **(2.83e-4)**
DTLZ7	3	22	Mean Std	2.0559e-1 (1.68e-2)+	2.2576e-1 (1.69e-2)+	**2.3047e-1** **(8.11e-3)+**	4.4636e-3 (2.44e-2)-	5.6708e-2 (3.61e-2)-	8.8424e-2 (2.57e-2)
Best/all				**2/22**	**5/22**	**1/22**	**0/22**	**0/22**	**13/22**
*- / + / ≈*				**13/5/4**	**11/8/3**	**13/7/2**	**17/1/4**	**17/2/3**	

### Problem-dependent performance analysis

To further explain the performance differences observed in Tables 3, 4, 5 and 6, a problem-dependent analysis is provided from the perspective of the characteristics of different benchmark problems and the mechanisms of TAMOPSO.

For ZDT4, the search space includes numerous local Pareto-optimal regions, making particles more susceptible to local optima. TAMOPSO’s advantage on this problem largely stems from its stagnation-detection-driven particle reconstruction strategy. Once a particle shows no improvement over several successive iterations, the reconstruction mechanism is triggered and sparsely distributed elite solutions in the external archive are used as guidance samples. This operation helps stagnant particles leave locally attractive but unproductive regions and return to promising search areas. In addition, the bounded auxiliary archive preserves discarded non-dominated solutions during archive maintenance, which provides additional historical guidance for the pbest update and reduces the risk of losing potentially useful search directions. Therefore, TAMOPSO is particularly effective for multimodal problems such as ZDT4.

For UF10, the problem involves a complex three-objective landscape with strong nonlinear interactions among decision variables. In this case, relying only on dominance-based pbest updating may easily weaken the search guidance because many candidate solutions become mutually non-dominated during evolution. TAMOPSO alleviates this issue through the auxiliary-archive-assisted pbest update mechanism. The discarded but still high-quality non-dominated solutions are reused to guide the update of personal best positions, thereby broadening the range of search directions. In addition, the two-stage archive maintenance strategy combines grid-based density identification, angle-based diversity evaluation, and dual-distance convergence assessment, enabling the archive to retain solutions with both convergence potential and distribution contribution. These mechanisms jointly explain the strong performance of TAMOPSO on complex UF problems, especially UF10.

By contrast, the relatively weaker performance of TAMOPSO on DTLZ2 and DTLZ4 is mainly related to the scalability of the current archive maintenance and guidance mechanisms in three-objective spaces. DTLZ2 has a regular concave spherical Pareto front, and the main difficulty lies not only in convergence but also in maintaining a uniformly distributed approximation over the entire two-dimensional manifold. Under a fixed archive capacity, mutually non-dominated solutions become much more numerous in three-objective spaces. As a result, grid-based density estimation and angle-based diversity evaluation may become less discriminative, and some useful boundary or intermediate solutions may be removed during archive maintenance. This may result in inadequate Pareto-front coverage, thereby influencing the IGD and HV results. DTLZ4 further increases this difficulty because its biased mapping tends to drive solutions toward certain regions of the Pareto front, leading to non-uniform solution distributions. Although the proposed archive maintenance strategy can delete crowded solutions and the reconstruction strategy can redirect stagnant particles, both the auxiliary archive and the reconstruction guidance samples are derived from the current external archive. Therefore, when the archive itself is biased or incomplete, the guidance information may also inherit this distributional bias. This limitation is mainly related to the fact that the proposed archive maintenance strategy relies on the quality of the current external archive to estimate density and diversity information. When the archive distribution becomes biased or insufficiently representative, the subsequent selection and guidance processes may inherit such bias.

This explains why TAMOPSO performs strongly on multimodal and complex-front problems such as ZDT4 and UF10, but shows relatively weaker performance on DTLZ2 and DTLZ4, where uniform coverage of a higher-dimensional Pareto front is more demanding.

### Analysis of pareto-front approximation results

**Fig. 7:**
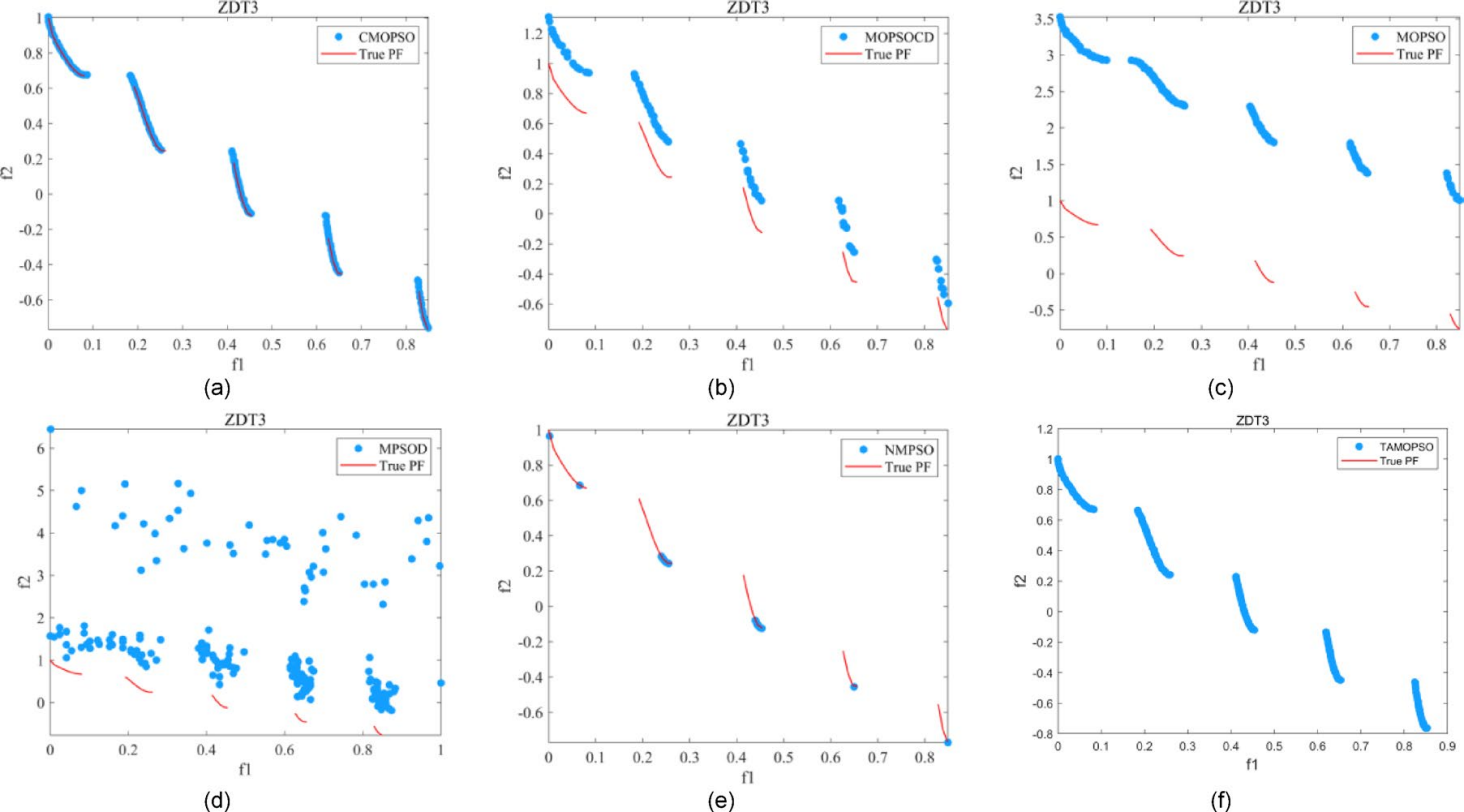
PF plots of five MOPSOs and TAMOPSO on ZDT3.

**Fig. 8:**
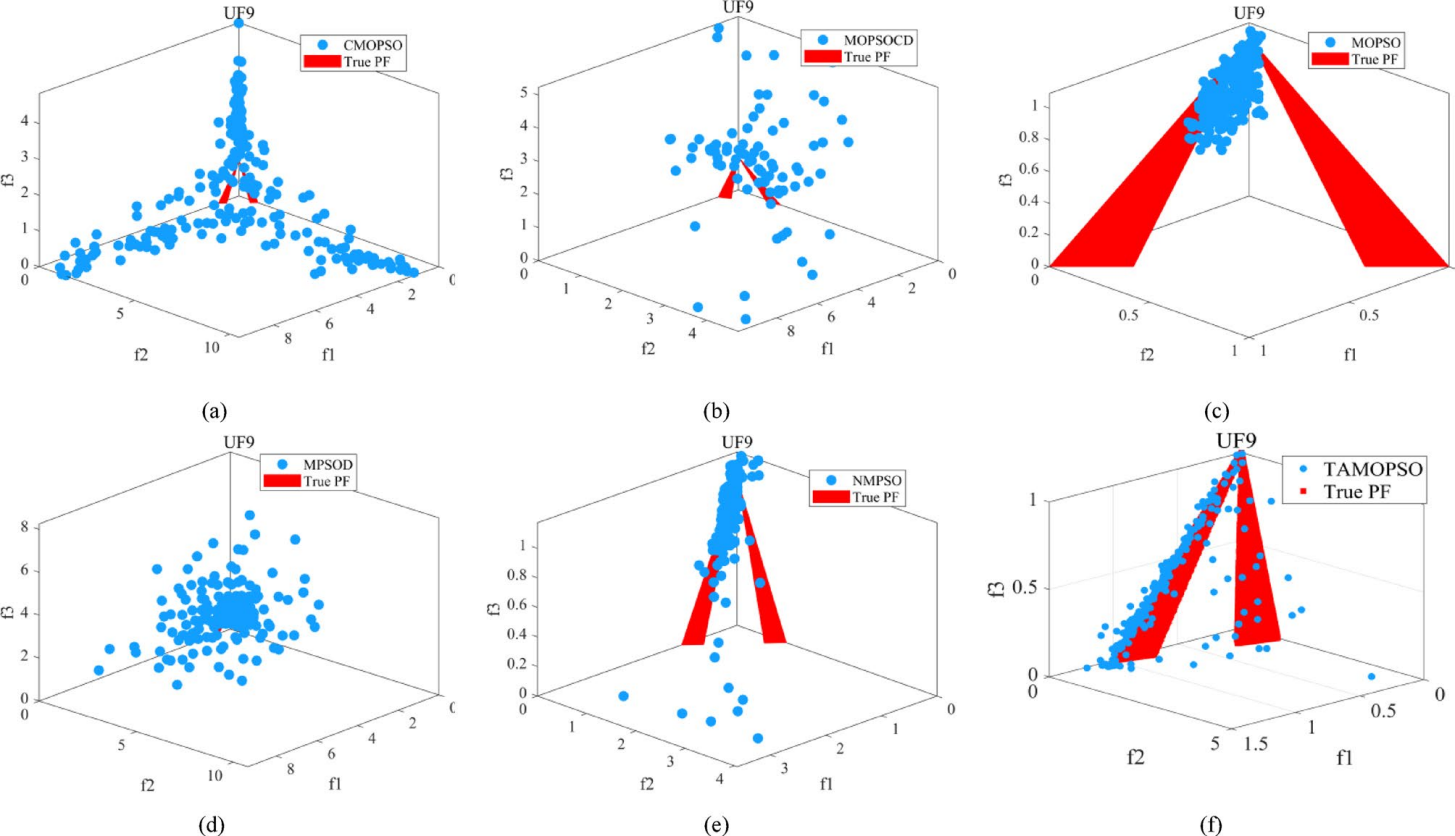
PF plots of five MOPSOs and TAMOPSO on UF9.

**Fig. 9:**
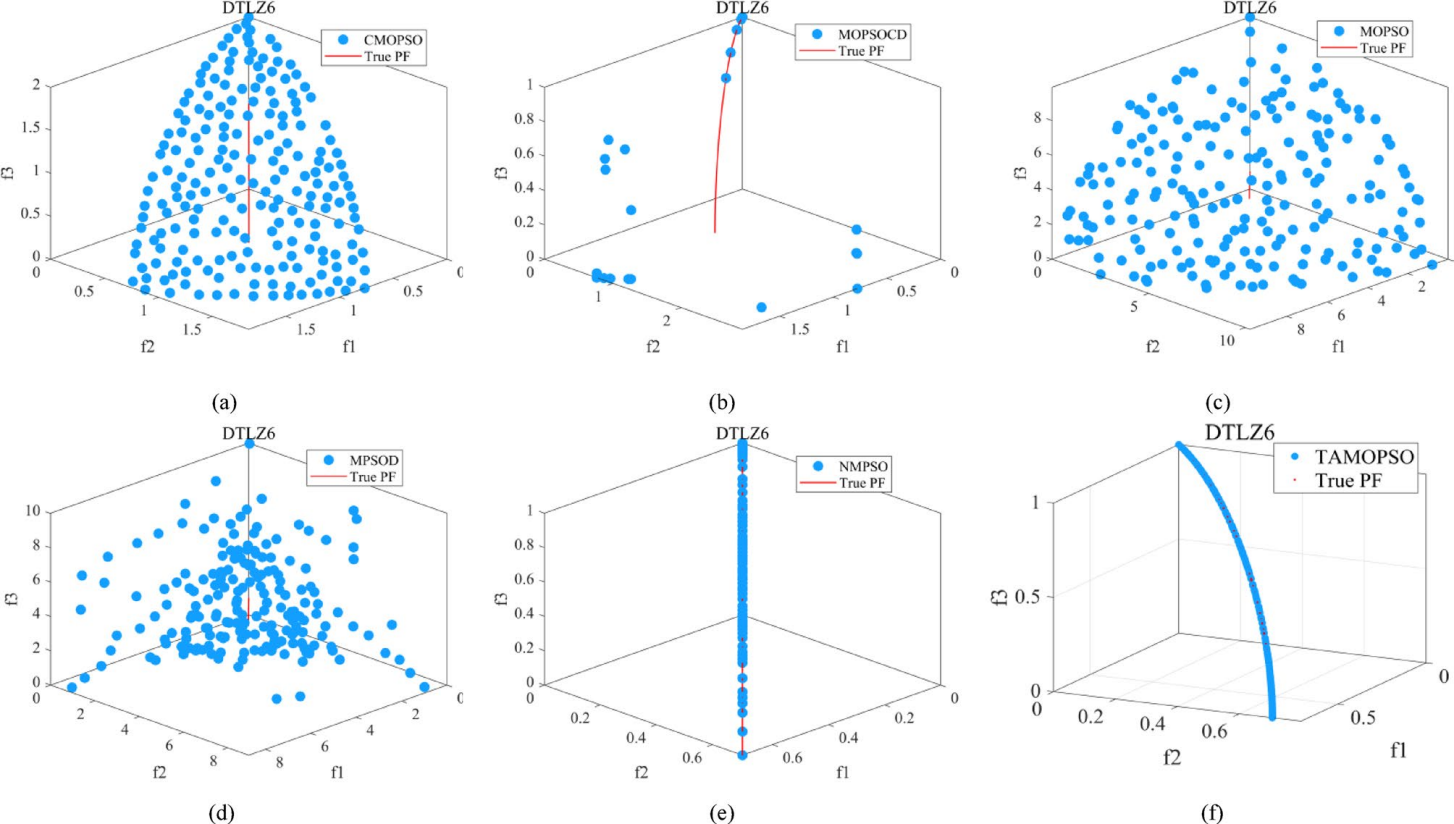
PF plots of five MOPSOs and TAMOPSO on DTLZ6.

Figures 7, 8 and 9 present the Pareto front diagrams for the TAMOPSO algorithm and five other MOPSO algorithms on the ZDT3, UF9, and DTLZ6 test problems, illustrating the fit between the Pareto fronts obtained by each algorithm and the true front. The figures reveal that the solution set obtained by TAMOPSO exhibits a substantial overlap with the true Pareto front compared with the other five algorithms, demonstrating notable convergence. Although the solutions do not uniformly cover the entire Pareto front, no significant clustering or gaps are discernible.

**Fig. 10:**
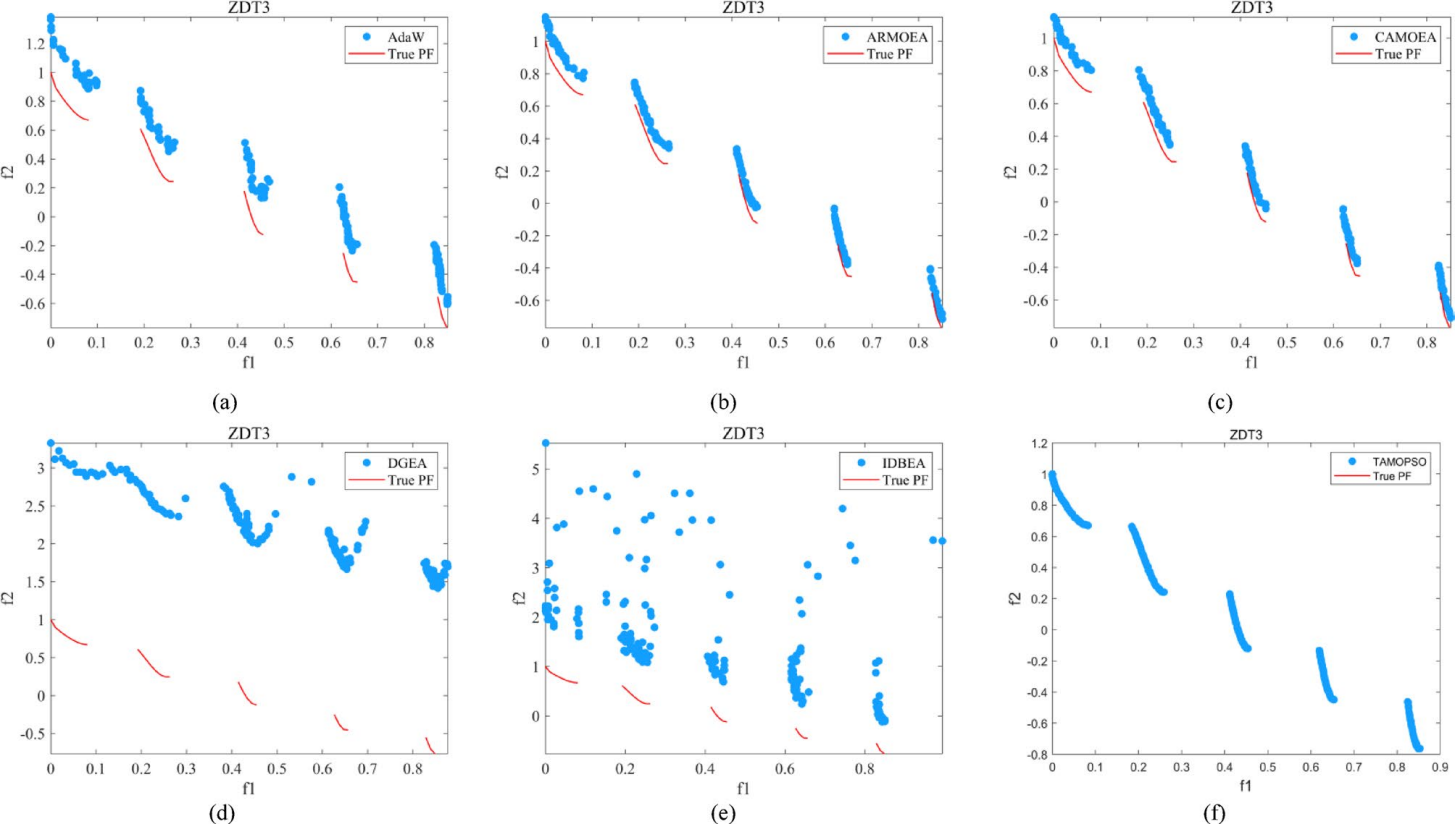
PF plots of five MOEAs and TAMOPSO on ZDT3.

**Fig. 11:**
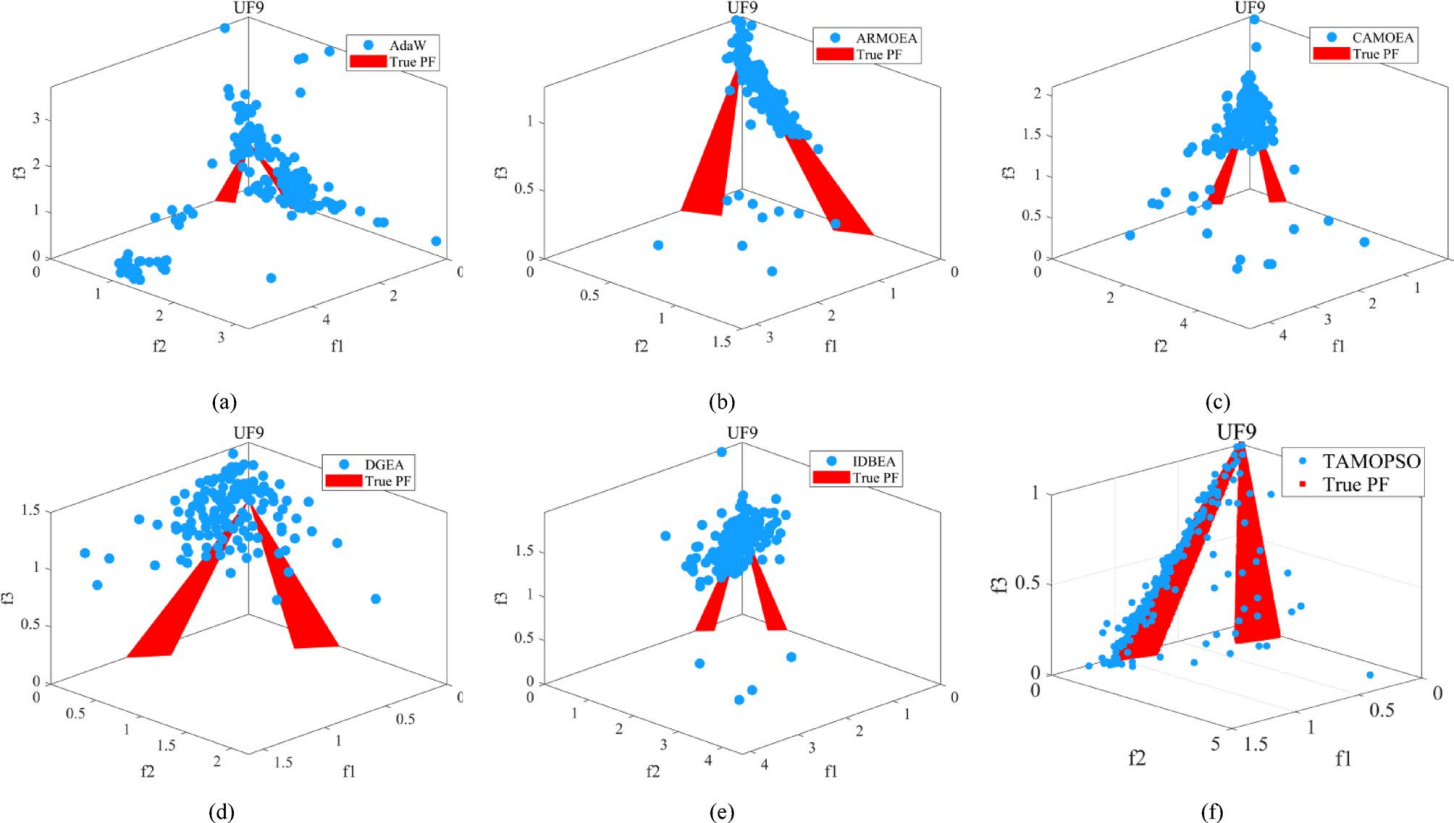
PF plots of five MOEAs and TAMOPSO on UF9.

**Fig. 12:**
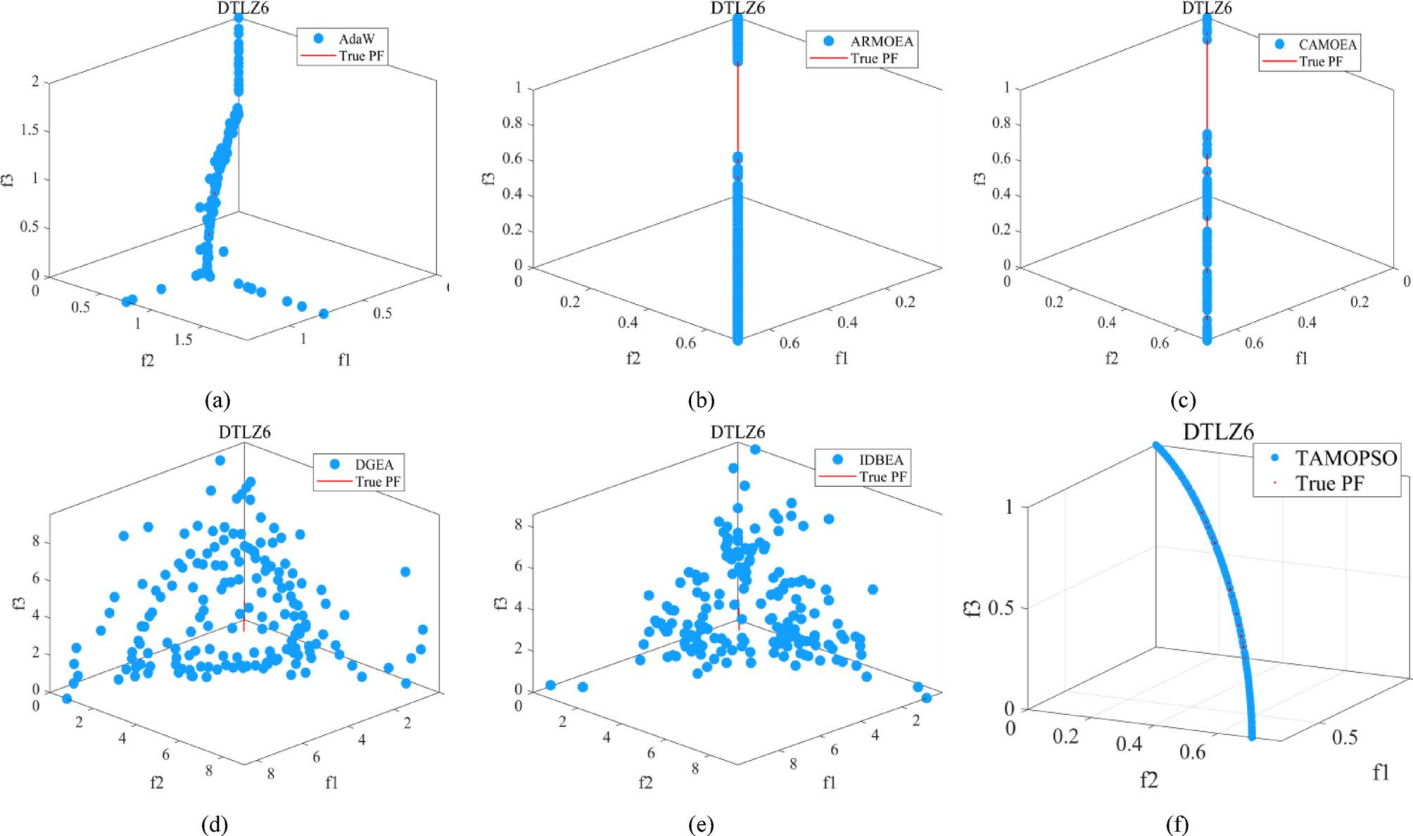
PF plots of five MOEAs and TAMOPSO on DTLZ.

Figures 10, 11, and 12 present the Pareto front plots for the TAMOPSO algorithm alongside the other five evolutionary algorithms for the same test problems. Regarding convergence, the blue scatter points of TAMOPSO closely aligned with the true PF, exhibiting only minor deviations at the curve discontinuities. Regarding the distribution, the scatter points of TAMOPSO were continuously and uniformly distributed along the true PF curve. The solution sets from the other comparison algorithms exhibited discrete distributions, with noticeable gaps between them and the true Pareto front. This indicates their failure to effectively converge to high-quality solution regions.

### Box plot results analysis

**Fig. 13:**
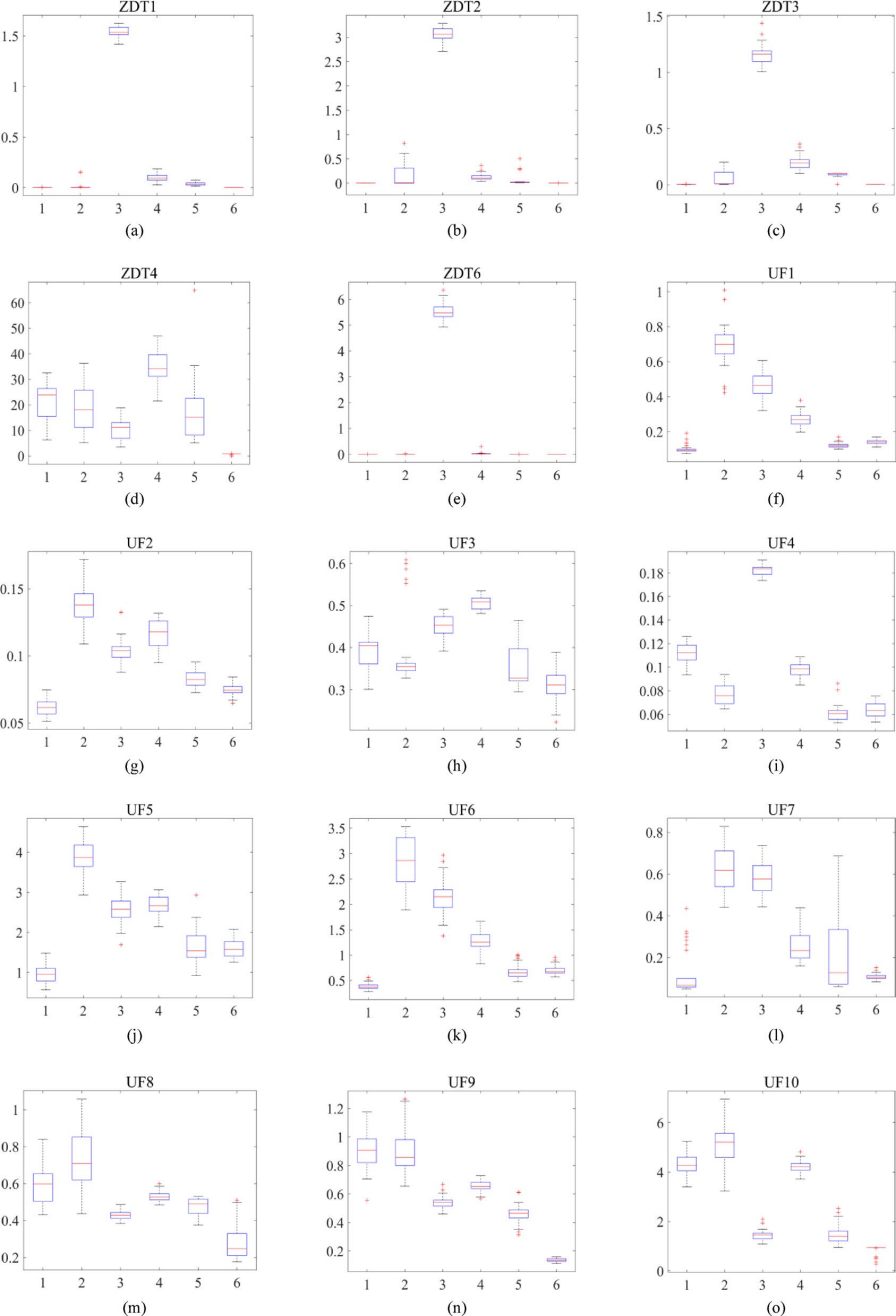
Box plots of IGD values for five MOPSOs and TAMOPSO on ZDT and UF series test functions.

**Fig. 14:**
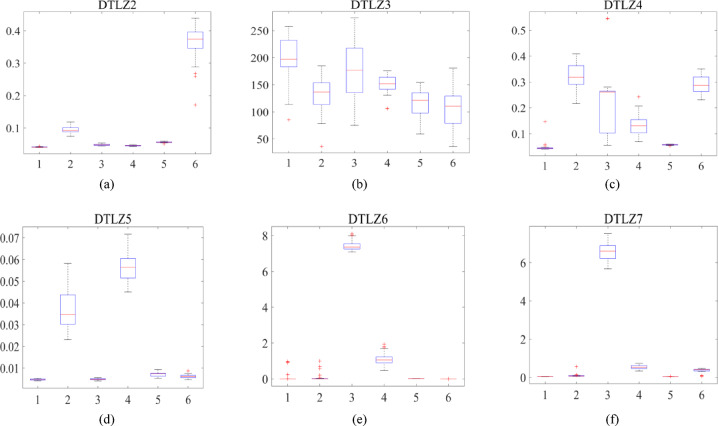
Box plots of IGD values for five MOPSOs and TAMOPSO on DTLZ series test functions.

Figures 13 and 14 present the IGD box plots for the TAMOPSO algorithm alongside five other MOPSO algorithms across the ZDT, UF, and DTLZ test problem series. In Figs. 13 and 14, the six boxes in each subfigure are arranged from left to right as CMOPSO, MOPSOCD, MOPSO, MPSOD, NMPSO, and TAMOPSO. For most test problems, the TAMOPSO algorithm’s IGD box plot occupies the lowest and most compact position, demonstrating the algorithm’s superior convergence, diversity, and robustness. In the DTLZ7 test problem, TAMOPSO still leads in convergence, but its box plot distribution range increases compared to problems such as DTLZ2. This indicates that the algorithm’s performance is insufficient when dealing with such discontinuous front structures, leaving room for improvement.

**Fig. 15:**
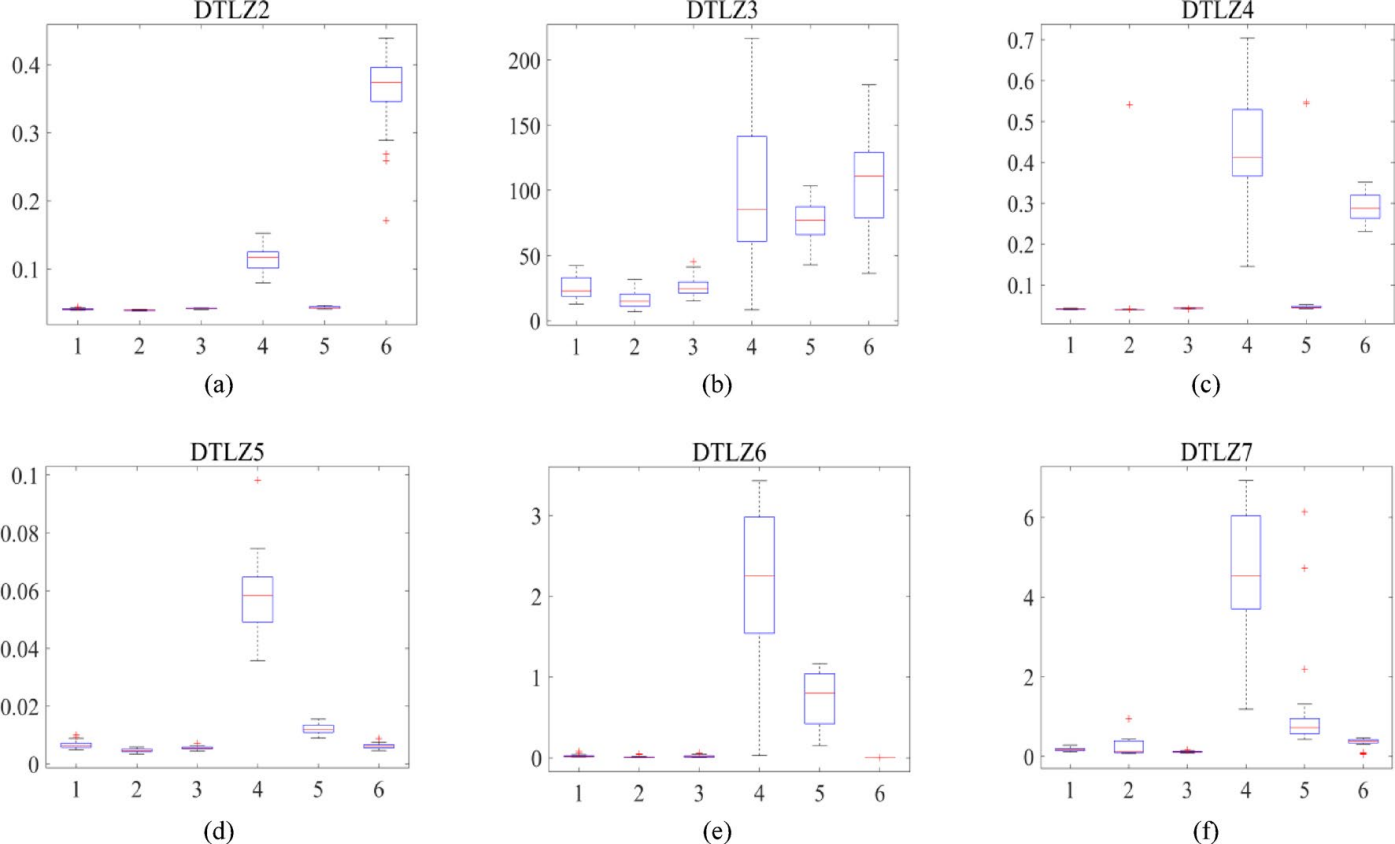
Box plots of IGD values for five MOEAs and TAMOPSO on DTLZ series test functions.

**Fig. 16:**
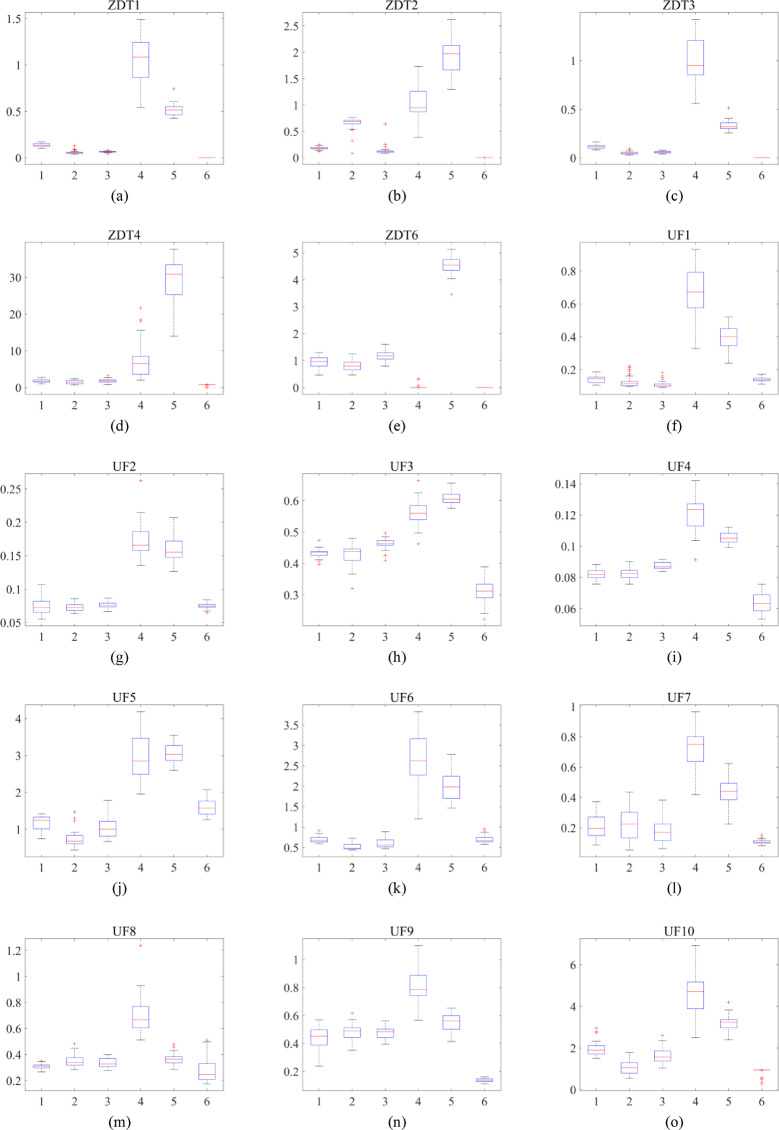
Box plots of IGD values for five MOEAs and TAMOPSO on ZDT and UF series test function.

Figures 15 and 16 present the IGD box plots of TAMOPSO alongside five other evolutionary algorithms on the same test problems. In Figs. 15 and 16, the six boxes in each subfigure are arranged from left to right as AdaW, ARMOEA, CAMOEA, DGEA, IDBEA, and TAMOPSO, respectively. Evidently, the box plots of TAMOPSO are positioned below those of the other algorithms for most problems, exhibiting a flatter shape and lower variance. This demonstrates not only high convergence accuracy but also strong stability in the repeated experiments. However, for the DTLZ2 and DTLZ4 problems, TAMOPSO’s performance of TAMOPSO was sub-optimal, further indicating that improvements are still needed when handling problems with high-dimensional objective space distributions.

### Analysis of convergence trajectory diagram results

**Fig. 17:**
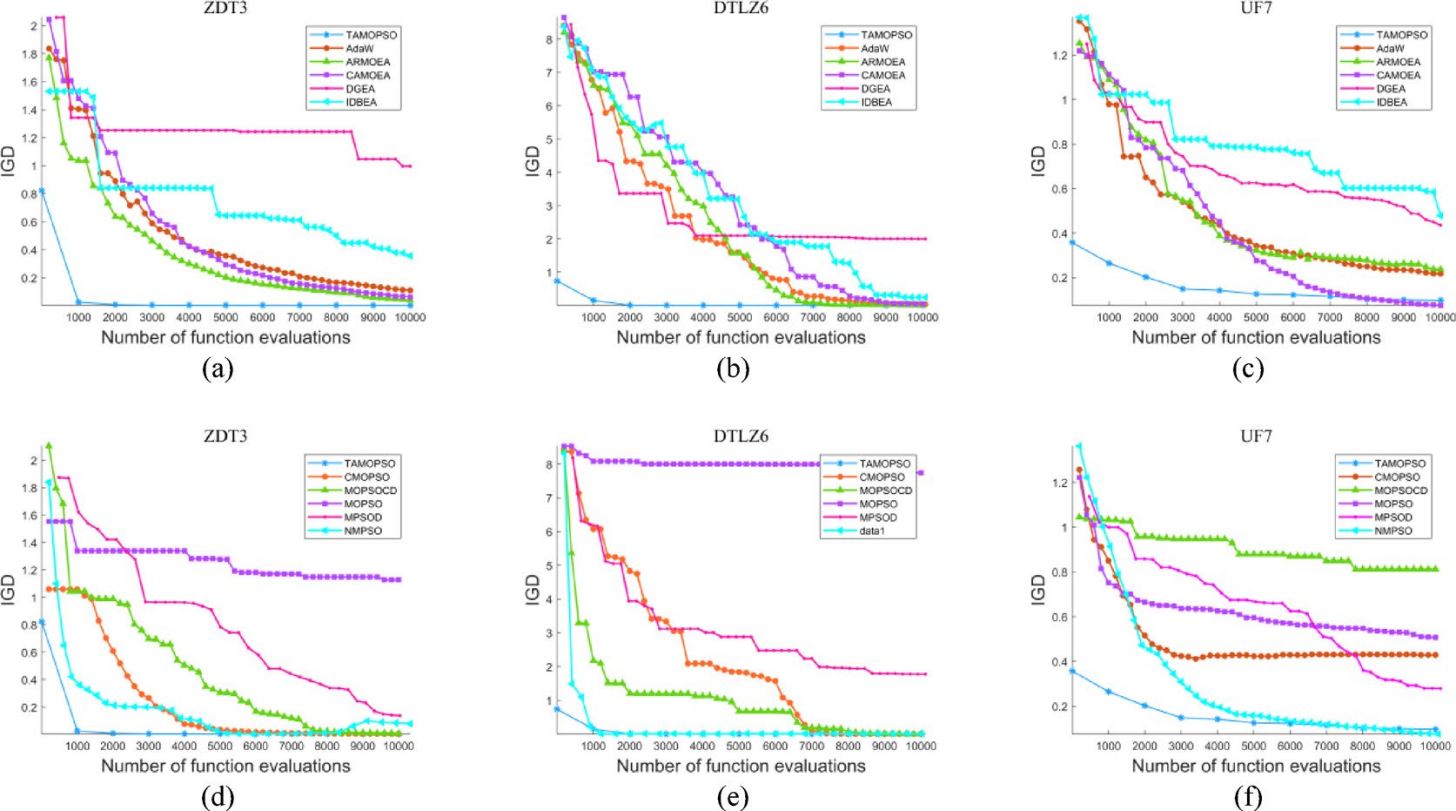
IGD convergence trajectories for 10 comparison algorithms and TAMOPSO on ZDT3 UF7, and DTLZ6 test functions.

Figure 17a–c depict the IGD convergence trajectories of TAMOPSO alongside five other evolutionary algorithms on the same test problems. In the convergence-curve figures, different colors and line styles represent different algorithms, as indicated in the legends. The same legend order is used consistently across all convergence plots.The trajectory plots reveal TAMOPSO’s substantial superiority of TAMOPSO over the comparison algorithms in terms of convergence speed and solution set quality. In contrast, although algorithms such as AdaW and ARMOEA are competitive, they exhibit deficiencies in convergence velocity and stability.

Figure 17d–f depict the IGD convergence trajectories of TAMOPSO alongside five other MOPSO algorithms for the ZDT3, UF7, and DTLZ7 test problems. TAMOPSO demonstrated a significant advantage across all tests, particularly for the most challenging DTLZ6 problem. While the trajectories of the comparison algorithms largely stagnated at high values, TAMOPSO was the sole algorithm capable of sustained optimization and effective convergence to a high-quality solution.

The above experiments conclusively demonstrate that the TAMOPSO algorithm not only effectively balances population convergence and diversity in multi-objective optimization problems but also exhibits outstanding algorithmic stability.

**Table 7:** Friedman test results of IGD values for all comparison algorithms and TAMOPSO. Significant values are in bold and italic.

Algorithm	ZDT1–4, ZDT6		UF1–10		DTLZ 1–7		Total	
	Friedman test	Rank	Friedman test	Rank	Friedman test	Rank	Friedman test	Rank
CMOPSO	3.2	2	4.9	6	5.43	5	4.68	6
MOPSOCD	4.6	3	9	10	8	9	7.68	7
MOPSO	10	11	7.4	7	7.86	8	8.14	10
MPSOD	7.2	8	7.7	8	8	9	7.68	7
NMPSO	4.6	3	4.2	3	5.14	4	4.59	5
AdaW	6.6	7	4.2	3	3.43	3	4.5	4
ARMOEA	5.2	5	3.5	2	2.57	1	3.59	1
CAMOEA	6	6	4.2	3	3.29	2	4.32	3
DGEA	7.8	9	10	11	9.29	11	9.27	11
IDBEA	9.6	10	8.2	9	6.14	6	7.86	9
TAMOPSO	1.2	**1**	2.7	**1**	6.86	*7*	3.68	2

**Table 8:** Friedman test results of HV values for all comparison algorithms and TAMOPSO.

Algorithm	ZDT1–4, ZDT6		UF1–10		DTLZ 1–7		Total	
	Friedman test	Rank	Friedman test	Rank	Friedman test	Rank	Friedman test	Rank
CMOPSO	2.9	2	4.55	4	4.79	5	4.25	4
MOPSOCD	4.5	4	8.1	9	7.36	8	7.05	7
MOPSO	10	11	7.7	7	7.79	9	8.25	10
MPSOD	6.7	7	7.7	7	8.36	10	7.68	8
NMPSO	4.3	3	4	3	3.36	1	3.86	2
AdaW	7.5	8	5.3	6	4.29	4	5.48	6
ARMOEA	5	5	3.55	2	3.71	2	3.93	3
CAMOEA	6.2	6	4.65	5	4	3	4.8	5
DGEA	8.3	9	9.2	11	8.57	11	8.8	11
IDBEA	9.2	10	8.3	10	7.21	7	8.16	9
TAMOPSO	1.4	1	2.95	1	6.57	6	3.75	1

### Analysis of Friedman test results

To statistically evaluate the overall ranking of the algorithms and examine whether the observed performance differences were significant, this study conducted Friedman tests on the performance of all comparison algorithms across the ZDT, UF, and DTLZ problem series. The test results for the IGD and HV metrics are presented in Tables 7 and 8 respectively.

Regarding the IGD metric (Table 7), TAMOPSO ranked second overall, demonstrating excellent overall convergence and distributional properties. TAMOPSO exhibited strong performance across both the ZDT (ranked 1 st) and UF (ranked 1 st) series, significantly outperforming the other comparison algorithms. This confirms its effectiveness in addressing diverse bi-objective problems. However, its relatively lower ranking in the DTLZ series (7th place) suggests that the distribution of the solution set remains insufficient when approximating the true Pareto front in three-objective problems. This represents a key area that requires future refinement.

Regarding the HV metric (Table 8), the TAMOPSO algorithm demonstrated superior overall performance, securing the top position in the comprehensive ranking. This indicates that TAMOPSO achieved the best overall HV ranking among all compared algorithms in terms of overall performance when evaluating the comprehensive quality of solution sets using the HV metric. Specifically, it maintained an absolute advantage in both the ZDT (ranked 1 st) and UF (ranked 1 st). Within the DTLZ series, its ranking (6th) surpasses its performance on IGD but remains outside the leading positions. This indicates that although the algorithm can locate some high-quality solutions in high-dimensional spaces, the completeness of the solution set requires further improvement.

The Friedman test results indicate that TAMOPSO has a statistically competitive advantage in bi-objective optimization in dual-objective optimization was statistically significant and generally superior to those of the other comparison algorithms. The performance bottleneck observed in the three-objective problems, particularly the DTLZ series, was also statistically significant, clearly delineating its strengths and areas requiring improvement.

It should be noted that the relatively lower ranking of TAMOPSO on the DTLZ series does not simply indicate the failure of the proposed strategies, but reflects the scalability limitation of the current archive maintenance and guidance mechanisms in higher-dimensional objective spaces. Compared with bi-objective problems, three-objective DTLZ problems usually generate a larger number of mutually non-dominated solutions, which increases the selection pressure on the external archive. Under a fixed archive capacity, some potentially useful solutions may be removed during archive maintenance, resulting in insufficient coverage of the Pareto front. In addition, the grid-based density estimation and angle-based diversity evaluation become less discriminative as the number of objectives increases, making it more difficult to accurately identify sparse and crowded regions. The auxiliary archive and particle reconstruction strategies can partially alleviate stagnation and improve exploration, but their guiding samples are still derived from the external archive; therefore, their effectiveness is also affected when the archive distribution is incomplete. These factors jointly explain why TAMOPSO achieves strong performance on ZDT and UF problems but shows relatively weaker statistical rankings on the three-objective DTLZ series.

### Wilcoxon signed-rank test

**Fig. 18:**
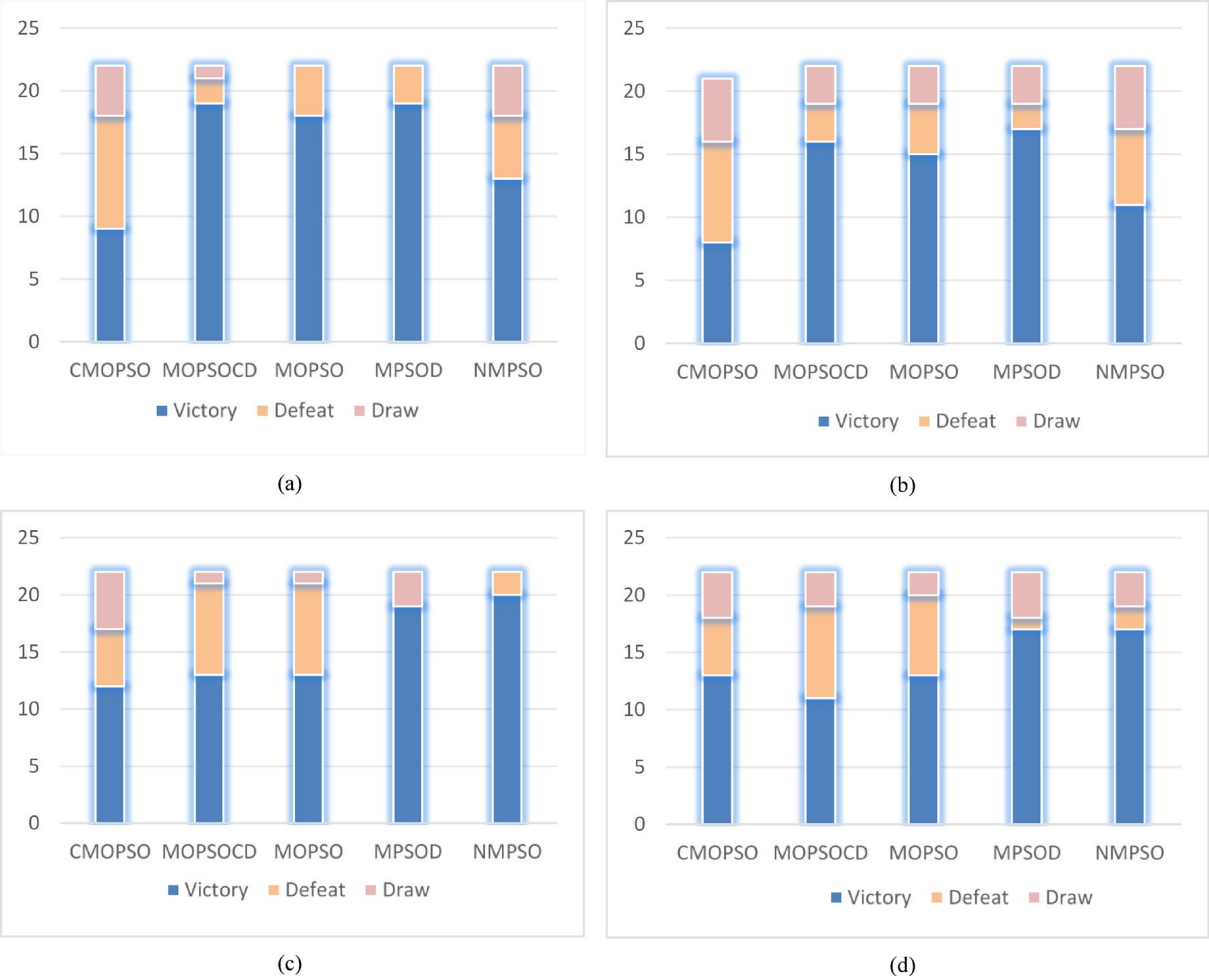
Wilcoxon signed-rank test results bar chart.

To validate the statistical significance of the algorithmic improvements, we employed Wilcoxon signed-rank tests to compare the performance of the proposed TAMOPSO algorithm with that of competing algorithms across multiple test datasets. The results are presented in Tables 2, 3, 4 and 5, where the symbols +, *\:-*, and $\:\approx\:$ denote instances where the comparison algorithm outperformed, underperformed, or matched the proposed algorithm on specific test functions, respectively. Figure 18 presents a bar chart illustrating the Wilcoxon signed-rank test results for TAMOPSO against various comparison algorithms across multiple test sets, categorizing the outcomes as “outperformed ”,“underperformed” or “tied”. The results show that TAMOPSO outperformed most comparison algorithms on a larger number of test instances. Thus, the Wilcoxon signed-rank test results statistically validated the efficacy and robustness of the TAMOPSO algorithm.

### Analysis of ablation experiment results

To validate the effectiveness of the three improvement strategies proposed in the TAMOPSO algorithm and their synergistic effects, systematic ablation experiments were designed. The purpose of the ablation study was to examine the effect of different component combinations and quantify the contribution of each proposed strategy to the overall performance to the overall performance of the algorithm, thereby demonstrating the rationality and necessity of the TAMOPSO algorithm design. Tables 9 and 10 report the ablation results in terms of IGD and HV, respectively.

TAMOPSO-23,TAMOPSO-13, and TAMOPSO-12 denote the variants obtained by removing Improvement 1, Improvement 2, and Improvement 3 from the complete TAMOPSO, respectively.

Improvement 1: External archive updates based on a two-stage maintenance strategy;

Improvement 2: The auxiliary-archive-based pbest update mechanism;

Improvement 3: The particle reconstruction strategy based on stagnation detection and archive guidance.

All algorithm variants were tested under identical conditions. Tables 9 and 10 show that, among the 22 test problems, TAMOPSO achieved the best IGD values on 15 problems and the best HV values on 13 problems. The number of best performances far exceeded that of any combination variant. Notably, for the DTLZ1 problem, where other algorithm variants failed to produce valid HV values, TAMOPSO was the sole algorithm that yielded a valid HV value.

**Table 9:** Ablation experiments on IGD. Bold indicates the best performance among TAMOPSO and its variant algorithms for each test problem.

Problem	M	D	IGD	TAMOPSO-23	TAMOPSO-13	TAMOPSO-12	TAMOPSO
ZDT1	2	30	Mean	5.55e-03	2.67e-03	3.11e-03	**2.61e-03**
			Std	5.62e-04	1.13e-04	1.56e-04	**1.13e-04**
ZDT2	2	30	Mean	1.44e-02	2.84e-03	2.95e-03	**2.81e-03**
			Std	4.79e-02	8.50e-05	1.21e-04	**1.91e-04**
ZDT3	2	30	Mean	5.68e-03	2.94e-03	3.28e-03	**2.91e-03**
			Std	1.67e-03	1.09e-04	2.43e-04	**1.76e-04**
ZDT4	2	10	Mean	7.85e-01	7.50e-01	7.85e-01	**7.42e-01**
			Std	2.08e-01	7.09e-02	1.68e-01	**2.54e-01**
ZDT6	2	10	Mean	1.63e-02	2.14e-03	**2.12e-03**	2.28e-03
			Std	4.54e-03	1.23e-04	**1.36e-04**	2.72e-04
UF1	2	30	Mean	1.41e-01	1.42e-01	1.47e-01	**1.38e-01**
			Std	1.41e-02	1.99e-02	1.33e-02	**1. 15e-02**
UF2	2	30	Mean	7.60e-02	7.58e-02	8.79e-02	**7.46e-02**
			Std	5.62e-03	2.78e-03	7.39e-03	**3.85e-03**
UF3	2	30	Mean	3.34e-01	3.21e-01	3.24e-01	**3.14e-01**
			Std	3.80e-02	3.78e-02	3.29e-02	**3.75e-02**
UF4	2	30	Mean	6.77e-02	6.82e-02	**6.35e-02**	6.39e-02
			Std	9.04e-03	8.54e-03	**4.18e-03**	5.93e-03
UF5	2	30	Mean	1.70e + 00	1.71e + 00	1.73e + 00	**1.61e + 00**
			Std	2.18e-01	1.81e-01	3.00e-01	**2.26e-01**
UF6	2	30	Mean	7.56e-01	7.26e-01	7.80e-01	**7.08e-01**
			Std	1.21e-01	9.71e-02	1.29e-01	**9.33e-02**
UF7	2	30	Mean	1.11e-01	1.11e-01	1.21e-01	**1.08e-01**
			Std	1.38e-02	1.73e-02	1.15e-02	**1.47e-02**
UF8	3	30	Mean	3.55e-01	3.09e-01	3.38e-01	**2.85e-01**
			Std	9.13e-02	1.02e-01	7.33e-02	**1.01e-01**
UF9	3	30	Mean	1.50e-01	**1.32e-01**	1.92e-01	1.37e-01
			Std	2.57e-02	**1.71e-02**	2.15e-02	1.19e-02
UF10	3	30	Mean	8.88e-01	**8.27e-01**	9.25e-01	8.63e-01
			Std	1.51e-01	**2.12e-01**	1.62e-01	1.89e-01
DTLZ1	3	7	Mean	1.59e + 01	1.59e + 01	1.86e + 01	**1.45e + 01**
			Std	4.70e + 00	4.10e + 00	3.96e + 00	**4.97e + 00**
DTLZ2	3	12	Mean	1.59e-01	4.82e-01	**1.37e-01**	3.61e-01
			Std	1.58e-02	9.61e-02	**9.05e-03**	6. 13e-02
DTLZ3	3	12	Mean	1.10e + 02	1.07e + 02	1.05e + 02	**1.05e + 02**
			Std	4.09e + 01	2.36e + 01	3.61e + 01	**3.51e + 01**
DTLZ4	3	12	Mean	2.95e-01	2.92e-01	3.47e-01	**2.88e-01**
			Std	3.59e-02	2.99e-02	2.85e-02	**3.26e-02**
DTLZ5	3	12	Mean	1.79e-02	**4.45e-03**	5.97e-02	6. 16e-03
			Std	7.77e-03	**2.87e-04**	9.71e-03	7.98e-04
DTLZ6	3	12	Mean	4.89e-03	3.03e-03	2.85e-03	**2.80e-03**
			Std	5.29e-04	1.60e-04	2.28e-04	**2.74e-04**
DTLZ7	3	22	Mean	**1.79e-01**	3.77e-01	2.80e-01	3.44e-01
			Std	**8.38e-02**	1.53e-02	9.26e-02	1.26e-01
Best/all				**1/22**	**3/22**	**3/22**	**15/22**

**Table 10:** Ablation experiments on HV. Bold indicates the best performance among TAMOPSO and its variant algorithms for each test problem.

Problem	M	D		TAMOPSO-23	TAMOPSO-13	TAMOPSO-12	TAMOPSO
ZDT1	2	30	Mean	7.16e-01	7.21e-01	7.20e-01	**7.21e-01**
			Std	1.22e-03	1.65e-04	2.75e-04	**1.62e-04**
ZDT2	2	30	Mean	4.46e-01	4.46e-01	4.45e-01	**4.62e-01**
			Std	9.99e-02	9.05e-05	2.75e-04	**2.08e-04**
ZDT3	2	30	Mean	5.98e-01	6.00e-01	6.00e-01	**6.00e-01**
			Std	1.64e-03	4.21e-04	9.22e-04	**2.16e-04**
ZDT4	2	10	Mean	1.32e-01	**1.80e-01**	1.30e-01	1.66e-01
			Std	1.55e-01	**6.63e-02**	1.09e-01	1.91e-01
ZDT6	2	10	Mean	3.77e-01	3.90e-01	3.90e-01	**4.01e-01**
			Std	3.87e-03	2.11e-04	1.39e-04	**1.49e-04**
UF1	2	30	Mean	5.02e-01	5.00e-01	4.91e-01	**5.03e-01**
			Std	2.33e-02	1.45e-02	2.09e-02	**1.90e-02**
UF2	2	30	Mean	6.34e-01	6.35e-01	6.19e-01	**6.35e-01**
			Std	5.30e-03	3.61e-03	7.67e-03	**2.85e-03**
UF3	2	30	Mean	3.21e-01	3.34e-01	3.24e-01	**3.44e-01**
			Std	2.77e-02	2.49e-02	2.27e-02	**3.54e-02**
UF4	2	30	Mean	3.50e-01	3.49e-01	**3.57e-01**	3.55e-01
			Std	1.13e-02	1.10e-02	**5.34e-03**	7.49e-03
UF5	2	30	Mean	0.00e + 00	0.00e + 00	0.00e + 00	0.00e + 00
			Std	0.00e + 00	0.00e + 00	0.00e + 00	0.00e + 00
UF6	2	30	Mean	4.85e-03	6.91e-03	3.66e-03	**8.32e-03**
			Std	7.32e-03	1.04e-02	6.94e-03	**3.03e-03**
UF7	2	30	Mean	4.17e-01	4.18e-01	4.03e-01	**4.22e-01**
			Std	2.01e-02	2.44e-02	1.63e-02	**2. 18e-02**
UF8	3	30	Mean	3.22e-01	3.40e-01	3.08e-01	**3.43e-01**
			Std	1.79e-02	2.86e-02	1.34e-02	**2.10e-02**
UF9	3	30	Mean	6.02e-01	6.20e-01	5.40e-01	**6.23e-01**
			Std	2.91e-02	2.42e-02	2.87e-02	**1.47e-02**
UF10	3	30	Mean	1.00e-01	1.09e-01	9.03e-02	**1.23e-01**
			Std	5.55e-02	3.48e-02	4.54e-02	**7.76e-02**
DTLZ1	3	7	Mean	0.00e + 00	0.00e + 00	0.00e + 00	**5.50e-03**
			Std	0.00e + 00	0.00e + 00	0.00e + 00	**2.96e-02**
DTLZ2	3	12	Mean	3.74e-01	2.12e-01	**3.96e-01**	2.74e-01
			Std	1.73e-02	4.32e-02	**1.67e-02**	3.46e-02
DTLZ3	3	12	Mean	0.00e + 00	0.00e + 00	0.00e + 00	0.00e + 00
			Std	0.00e + 00	0.00e + 00	0.00e + 00	0.00e + 00
DTLZ4	3	12	Mean	3.31e-01	**3.53e-01**	2.26e-01	3.50e-01
			Std	4.45e-02	**2.83e-02**	3.78e-02	4.23e-02
DTLZ5	3	12	Mean	1.82e-01	**1.99e-01**	1.32e-01	1.97e-01
			Std	1.11e-02	**3.35e-04**	1.28e-02	6.77e-04
DTLZ6	3	12	Mean	**3.57e-01**	2.01e-01	2.01e-01	2.01e-01
			Std	**3.21e-03**	3.36e-04	3.16e-04	2.83e-04
DTLZ7	3	22	Mean	**1.24e-01**	9.53e-02	9.70e-02	8.84e-02
			Std	**1.32e-02**	1.39e-02	2.23e-02	2.57e-02
Best/all				**2/22**	**3/22**	**2/22**	**13/22**

Tables 9 and 10 demonstrate that removing any single improvement strategy led to a clear performance degradation, indicating that all the proposed algorithmic enhancements are effective. The specific analyses are as follows:

The results of TAMOPSO-23, which excludes the two-stage archive maintenance strategy, show that this component is essential for maintaining both convergence and diversity. In particular, for the ZDT series problems, the IGD values were markedly inferior to those of the full TAMOPSO implementation. For instance, on ZDT2, TAMOPSO-23’s IGD value was an order of magnitude higher than that of TAMOPSO. This indicates that Improvement 1 is crucial for maintaining the solution set diversity and convergence.

Observing the results from TAMOPSO-13 (lacking Improvement 2: $\:\mathrm{p}\mathrm{b}\mathrm{e}\mathrm{s}\mathrm{t}$collaborative update), this variant demonstrated markedly sub-optimal performance on complex problems. On challenging test problems, such as UF5 and UF6, its IGD values were consistently worse than those of the full TAMOPSO algorithm. This demonstrates that Improvement 2 effectively guides particle trajectories by leveraging historical information from auxiliary archives, preventing particles from persistently wandering in unproductive regions, and thereby enhancing the search efficiency of the algorithm.

The performance analysis of TAMOPSO-12 (lacking Improvement 3: particle reconstruction strategy) indicates that this variant performs poorly when addressing multi-modal problems. For the DTLZ7 problem, TAMOPSO-12’s IGD value is markedly inferior to that of the full TAMOPSO, indicating that Improvement 3 enhances the ability of TAMOPSO to escape local optima by identifying and reconstructing stagnant particles. This is crucial for managing complex Pareto fronts.

Removing each component generally degraded the overall performance across the complete benchmark set, although some reduced variants outperformed the full algorithm on individual problems.

### Analysis of average runtime results

**Table 11:** Average runtime comparison of TAMOPSO and five MOPSO algorithms over 30 independent runs.

Problem		CMOPSO	MOPSOCD	MOPSO	MPSOD	NMPSO	TAMOPSO
ZDT1	Mean	1.17e + 00	2.23e-01	2.00e-01	1.26e + 00	5.56e + 00	2.03e + 00
ZDT2	Mean	9.89e-01	2.30e-01	2.33e-01	1.36e + 00	6.63e + 00	4.16e-01
ZDT3	Mean	5.06e-01	2.07e-01	1.93e-01	1.37e + 00	4.34e + 00	6.68e-01
ZDT4	Mean	5.79e-01	1.54e-01	2.15e-01	1.29e + 00	1.65e + 00	4.04e-01
ZDT6	Mean	2.37e + 00	2.63e-01	2.10e-01	1.29e + 00	8.01e + 00	5.30e-01
UF1	Mean	3.54e-01	2.03e-01	2.47e-01	1.01e + 00	3.38e-01	2.53e-01
UF2	Mean	2.82e-01	2.11e-01	2.77e-01	1.58e + 00	1.14e + 00	6.48e-01
UF3	Mean	3.92e-01	2.64e-01	2.59e-01	1.78e + 00	1.26e + 00	5.02e-01
UF4	Mean	3.96e-01	1.85e-01	1.37e-01	1.05e + 00	2.92e + 00	6.31e-01
UF5	Mean	3.29e-01	1.96e-01	2.49e-01	1.65e + 00	5.73e-01	3.95e-01
UF6	Mean	2.05e-01	1.36e-01	1.31e-01	1.22e + 00	4.42e-01	2.61e-01
UF7	Mean	4.42e-01	2.41e-01	1.22e-01	8.44e-01	4.50e-01	8.87e-01
UF8	Mean	8.09e-01	1.41e-01	4.06e-01	2.58e + 00	2.14e + 00	7.51e-01
UF9	Mean	9.31e-01	1.24e-01	3.50e-01	2.36e + 00	1.35e + 00	8.39e-01
UF10	Mean	5.55e-01	2.11e-01	2.60e-01	1.40e + 00	6.21e-01	6.20e-01
DTLZ1	Mean	2.59e-01	1.64e-01	2.24e-01	1.27e + 00	8.08e-01	3.82e-01
DTLZ2	Mean	3.54e + 00	1.81e-01	3.25e-01	1.31e + 00	5.69e + 00	3.13e + 00
DTLZ3	Mean	2.92e-01	1.85e-01	2.22e-01	1.35e + 00	9.99e-01	3.40e-01
DTLZ4	Mean	2.61e + 00	1.66e-01	2.76e-01	1.28e + 00	5.33e + 00	3.15e-01
DTLZ5	Mean	1.62e + 00	1.84e-01	3.09e-01	1.46e + 00	2.67e + 00	1.94e + 00
DTLZ6	Mean	1.61e + 00	2.54e-01	2.87e-01	1.52e + 00	8.39e + 00	1.98e + 00
DTLZ7	Mean	1.57e + 00	2.10e-01	2.67e-01	1.35e + 00	5.06e + 00	6.84e-01

**Table 12:** Average runtime comparison of TAMOPSO and five MOEAs algorithms over 30 independent runs.

Problem		AdaW	ARMOEA	CAMOEA	DGEA	IDBEA	TAMOPSO
ZDT1	Mean	3.16e + 00	8.84e-01	3.23e-01	4.83e-01	3.99e + 00	2.03e + 00
ZDT2	Mean	3.06e + 00	4.07e-01	2.97e-01	8.44e-01	4.26e + 00	4.16e-01
ZDT3	Mean	3.55e + 00	9.94e-01	2.53e-01	4.17e-01	4.54e + 00	6.68e-01
ZDT4	Mean	3.02e + 00	3.00e-01	2.18e-01	4.18e-01	3.99e + 00	4.04e-01
ZDT6	Mean	6.67e + 01	3.35e-01	1.82e-01	4.15e-01	4.12e + 00	5.30e-01
UF1	Mean	3.48e + 00	4.71e-01	1.75e-01	3.77e-01	6.48e + 00	2.53e-01
UF2	Mean	4.97e + 00	1.16e + 00	3.37e-01	6.06e-01	6.30e + 00	6.48e-01
UF3	Mean	4.78e + 00	5.71e-01	2.84e-01	5.07e-01	4.77e + 00	5.02e-01
UF4	Mean	4.46e + 00	1.29e + 00	3.35e-01	5.41e-01	5.70e + 00	6.31e-01
UF5	Mean	3.98e + 00	4.27e-01	2.99e-01	4.88e-01	5.75e + 00	3.95e-01
UF6	Mean	4.47e + 00	4.33e-01	3.05e-01	4.87e-01	5.23e + 00	2.61e-01
UF7	Mean	3.82e + 00	6.60e-01	2.82e-01	5.10e-01	4.91e + 00	8.87e-01
UF8	Mean	5.43e + 00	2.44e + 00	3.75e-01	4.84e-01	5.45e + 00	7.51e-01
UF9	Mean	5.08e + 00	1.97e + 00	3.97e-01	4.97e-01	5.37e + 00	8.39e-01
UF10	Mean	5.16e + 00	1.30e + 00	3.60e-01	5.21e-01	5.64e + 00	6.20e-01
DTLZ1	Mean	3.54e + 00	6.89e-01	2.65e-01	4.22e-01	5.38e + 00	3.82e-01
DTLZ2	Mean	1.11e + 01	1.13e + 01	4.64e-01	4.98e-01	6.69e + 00	3.13e + 00
DTLZ3	Mean	3.76e + 00	5.29e-01	3.05e-01	5.28e-01	4.69e + 00	3.40e-01
DTLZ4	Mean	1.11e + 01	8.24e + 00	4.27e-01	4.15e-01	1.05e + 01	3.15e-01
DTLZ5	Mean	8.92e + 00	8.72e + 00	4.41e-01	4.02e-01	8.59e + 00	1.94e + 00
DTLZ6	Mean	5.92e + 00	6.14e + 00	3.94e-01	5.15e-01	5.98e + 00	1.98e + 00
DTLZ7	Mean	5.56e + 00	4.26e + 00	1.89e-01	4.31e-01	5.40e + 00	6.84e-01

To assess the computational efficiency of the compared algorithms, Tables 11 and 12 present the average runtime over 30 independent executions for each test problem. All algorithms were tested under identical experimental conditions, including an equal maximum number of function evaluations. As shown in the table, although TAMOPSO introduces additional improvement strategies, its runtime is still comparable in magnitude to those of the compared MOPSO variants. This indicates that the proposed algorithm maintains an acceptable computational cost while enhancing the overall optimization performance.

### Parameter sensitivity analysis

Tables 13 and 14 show that TAMOPSO achieves the best overall performance when the auxiliary archive capacity is set to $\:{N}_{aux}=N$. A smaller archive preserves insufficient historical non-dominated information and weakens the guidance for *\:pbest* updating, whereas an excessively large archive may introduce redundant information and reduce guidance quality. Therefore, $\:{N}_{aux}=N$ provides a reasonable balance among historical information utilization, population diversity, and computational cost. Although (1.5 N) yields a slightly better IGD value on DTLZ2, its HV value decreases noticeably, indicating that both convergence and distribution should be considered in parameter selection.

**Table 13:** IGD results with different auxiliary archive sizes (*N* = 200).

Problem	0.25 *N*	0.5 *N*	*N*	1.5 *N*	2 *N*
ZDT1	3.0824e-03	2.8246e-03	2.6717e-03	2.7361e-03	3.8925e-03
ZDT3	3.4118e-03	3.1027e-03	2.9266e-03	2.9742e-03	3.1538e-03
ZDT4	9.1265e-01	8.0347e-01	7.4174e-01	7.6932e-01	8.2568e-01
UF7	2.2874e-01	1.1542e-01	1.0760e-01	1.5038e-01	2.1765e-01
DTLZ2	4.0326e-01	3.984e-01	3.6070e-01	3.2841e-01	4.8627e-01
DTLZ7	4.2135e-01	3.7486e-01	3.4423e-01	3.9884e-01	4.1271e-01

**Table 14:** HV results with different auxiliary archive sizes(*N* = 200).

Problem	0.25 *N*	0.5 *N*	*N*	1.5 *N*	2 *N*
ZDT1	6.6586e-01	6.9532e-01	7.2141e-01	7.0064e-01	6.6847e-01
ZDT3	5.8984e-01	5.9672e-01	6.0026e-01	5.9931e-01	5.9588e-01
ZDT4	1.1874e-01	1.4852e-01	1.6632e-01	1.5847e-01	1.3985e-01
UF7	3.9184e-01	4.1276e-01	4.2245e-01	4.1867e-01	4.0793e-01
DTLZ2	2.0163e-01	2.3695e-01	2.7373e-01	2.1048e-01	1.9317e-01
DTLZ7	6.9840e-02	8.1270e-02	8.8424e-02	8.4910e-02	7.7360e-02

Tables 15 and 16 indicate that a stagnation threshold of 3 produces the best IGD and HV results on all tested problems. A smaller threshold may trigger particle reconstruction too frequently and disturb the normal search process, whereas a larger threshold may delay the recovery of genuinely stagnant particles. Therefore, a threshold of 3 achieves an appropriate balance between avoiding false reconstruction and responding promptly to search stagnation. Overall, $\:{N}_{aux}=N$ and a stagnation threshold of 3 provide the most stable overall performance.

**Table 15:** IGD results with different stagnation thresholds.

Problem	$\:\boldsymbol{\uptau\:}=$1	$\:\boldsymbol{\uptau\:}=$2	$\:\boldsymbol{\uptau\:}=3$	$\:\boldsymbol{\uptau\:}=$5	$\:\boldsymbol{\uptau\:}=$7
ZDT1	3.4415e-03	3.0496e-03	2.6717e-3	2.7442e-03	4.0036e-03
ZDT3	3.2768e-03	3.7815e-03	2.9266e-3	2.9884e-03	3.8689e-03
ZDT4	9.4821e-01	8.1256e-01	7.4174e-1	7.8629e-01	8.7435e-01
UF7	1.3016e-01	1.1628e-1	1.0760e-1	1.6189e-01	2.2057e-01
DTLZ2	4.0183e-01	3.7715e-01	3.6070e-1	3.6958e-01	4.4892e-01
DTLZ7	4.3657e-01	3.8214e-01	3.4423e-1	3.6625e-01	4.6376e-01

**Table 16:** HV results with different stagnation thresholds.

Problem	$\:\boldsymbol{\uptau\:}=$1	$\:\boldsymbol{\uptau\:}=$2	$\:\boldsymbol{\uptau\:}=3$	$\:\boldsymbol{\uptau\:}=$5	$\:\boldsymbol{\uptau\:}=$7
ZDT1	7.1726e-01	7.1988e-01	7.2141e-1	7.0051e-01	6.9863e-01
ZDT3	5.5218e-01	5.9781e-01	6.0026e-1	5.3892e-01	5.2317e-01
ZDT4	1.1042e-01	1.4586e-01	1.6632e-1	1.5371e-01	1.3264e-01
UF7	3.8873e-01	4.1182e-01	4.2245e-1	4.1638e-01	4.0257e-01
DTLZ2	2.4872e-01	2.6618e-01	2.7373e-1	2.0286e-01	2.2041e-01
DTLZ7	6.5830e-02	7.9640e-02	8.8424e-2	8.3150e-02	7.4220e-02

### Limitations and future work

On the three-objective DTLZ series, TAMOPSO obtained relatively lower IGD and HV rankings than on the ZDT and UF series. This suggests that the current archive maintenance and guidance mechanisms still have limited scalability when handling higher-dimensional objective spaces. In particular, the fixed archive capacity may restrict the coverage of a large number of non-dominated solutions, and the discriminative ability of grid-based density estimation and angle-based diversity evaluation may decrease as the number of objectives increases. We will focus on improving the scalability of TAMOPSO by developing many-objective archive maintenance strategies, adaptive archive capacity control, and more robust diversity estimation mechanisms for high-dimensional objective spaces.

The practical applicability of TAMOPSO has not yet been validated in real-world engineering scenarios. Future work will investigate its performance on representative engineering optimization problems.

## Conclusions

This study proposed TAMOPSO, a multi-objective particle swarm optimization algorithm integrating two-stage external archive maintenance, auxiliary-archive-assisted cognitive guidance, and stagnation-detection-based particle reconstruction. Experiments on the ZDT, UF, and DTLZ benchmark suites showed that TAMOPSO achieved competitive overall IGD and HV performance, with particularly strong results on the ZDT and UF problems. The ablation, runtime, and parameter sensitivity analyses further supported the complementary roles of the three proposed mechanisms. Nevertheless, the relatively weaker results on several three-objective DTLZ problems indicate that archive scalability and Pareto-front coverage require further improvement.

## Data Availability

All data produced and examined in this study are presented within the published article.
